# On the Security of Cell-Free Massive MIMO Networks

**DOI:** 10.3390/s26020353

**Published:** 2026-01-06

**Authors:** Hanaa Mohammed, Roayat I. Abdelfatah, Nancy Alshaer, Mohamed E. Nasr, Asmaa M. Saafan

**Affiliations:** 1 Electronics and Electrical Communications Engineering Department, Faculty of Engineering, Tanta University, Tanta 31527, Egypt; r.elseidy@psau.edu.sa (R.I.A.); n.a.alshaer@f-eng.tanta.edu.eg (N.A.); mohamed.nasr@f-eng.tanta.edu.eg (M.E.N.); 2 Electrical Engineering Department, College of Engineering, Prince Sattam Bin Abdulaziz University, Al-Kharj 16278, Saudi Arabia; 3 Network Planning Department, National Telecommunication Institute, Cairo 11768, Egypt; asmaa.safan@nti.sci.eg

**Keywords:** cell-free architecture, physical layer security, authentication, key management, secure handover, block coordinate descent

## Abstract

The rapid growth of wireless devices, the expansion of the Internet of Things, and the aggregate demand for Ultra-Reliable Low-Latency communications (URLLC) are driving the improvement of next-generation wireless systems. One promising emerging technology in this area is cell-free massive Multiple Input Multiple Output (maMIMO) networks. The distributed nature of Access Points presents unique security challenges that must be addressed to unlock their full potential. This paper studies the key security concerns in Cell Free Massive MIMO (CFMM) networks, including eavesdropping, Denial-of-Service attacks, jamming, pilot contamination, and methods for enhancing Physical Layer Security (PLS). We also provide an overview of security solutions specifically designed for CFMM networks and introduce a case study of a Reconfigurable Intelligent Surface (RIS)-aided secure scheme that jointly optimizes the RIS phase shifts with the artificial noise (AN) covariance under power constraints. The non-convex optimization problem is solved via the block coordinate descent (BCD) alternating optimization scheme. The combined RIS, AN, and beamforming configuration achieves a balanced trade-off between security and energy performance, resulting in moderate improvements over the individual schemes.

## 1. Introduction

The development of next-generation networks [1,2] has led to an increasing demand for hypermobile connectivity with higher data rates, higher connection density, low latency, and improved security, which transcend the capabilities of existing cellular networks. Next-generation networks are poised to support advanced technologies; however, ensuring privacy, security and operational efficiency presents significant challenges, with trust being a critical element in addressing these issues. Massive MIMO (maMIMO) technology [3,4] has been proposed to enhance the spectral and energy efficiency of cellular systems through straightforward processing. maMIMO is set to enhance user experience and enable new mobile services, remaining a key technology for both developed and emerging markets over the next decade [5]. Spatial multiplexing, spatial diversity, and beamforming are transmission methods offered by the MIMO system in the channel [6]. Channel hardening occurs when the number of antennas increases; the wireless channels behave more like a deterministic channel rather than a fading one, ensuring unpredictable behavior [4].

However, the deployment of numerous distributed antenna systems (DAS), commonly referred to as cell-free maMIMO (CFMM) or distributed MIMO, raises concerns about how to effectively combine high-bandwidth spectrum with massive connectivity [7]. Using MIMO in next-generation wireless networks faces some challenges, including propagation loss issues, hardware cost, and algorithmic complexities, as well as high power consumption, channel estimation, and feedback overhead, in addition to security issues [8]. A key security challenge in maMIMO systems [8] is the heightened risk of physical layer attacks. These include pilot contamination, which disrupts channel estimation and data integrity, as well as difficulties in distinguishing and mitigating unlicensed access and malicious activities due to the expanded attack surface [9]. The estimation error can converge to zero by utilizing large-scale arrays and independent channel paths. The number of independently measured scattered paths can increase linearly with the area of coverage. By increasing the number of antennas, inter-user interference can average out and become negligible due to the randomness in phase shifts [9].

CFMM is an essential technology expected to meet the growing demands by providing seamless coverage and improving spectral efficiency (SE). It does this by deploying many dispersed access points (APs) that can serve multiple users simultaneously, without the constraints of cell boundaries. The main benefits of CFMM include improved coverage, high SE, enhanced reliability, and consistent quality of service (QoS). Decreasing both spatial correlation and average path losses through the successful reduction in broadcast distances is another benefit of CFMM systems [10]. While CFMM networks are less resilient to active attacks than co-located maMIMO [11], which also offers promising performance improvements, they introduce new security challenges due to their decentralized architecture, dense deployment, and increased complexity. Table 1 presents a selection of papers related to the challenges and requirements of next-generation networks (CFMM is one of the futuristic technologies of the next generation), outlining their core research contributions and providing brief descriptions.

Recently, several randomness-driven pilot assignment algorithms have been proposed and demonstrated to provide strong performance, even in environments with only a few dominant paths or partially deterministic propagation. In particular, ref. [25] introduced a game-theoretical, randomness-based pilot selection framework that effectively mitigates pilot contamination in crowded time division duplex (TDD) maMIMO systems. Owing to its robustness, scalability, and applicability to practical deployment scenarios, this approach is highly promising, widely applicable, and is anticipated to receive increasing attention in the field. Although CFMM systems offer large coverage areas and reduced interference, scaling them in a frequency division duplex (FDD) setting [26] presents challenges due to high computational complexity for tasks like estimating the channel and the power distribution. Authors of [26] proposed a scalable FDD-based cell-free network with angular reciprocity and dynamic cooperation clustering (DCC), showing through simulations that it outperforms conventional schemes, providing significant improvements in spectral and energy efficiency.

This paper examines the security challenges and summarizes proposed strategies to mitigate them. It explores the threats, vulnerabilities, and countermeasures necessary for ensuring secure communication in CFMM systems. The main contribution of the paper includes (1) A comprehensive survey of security in CFMM networks, and (2) the case study and evaluation of a specific RIS-aided framework designed to address identified gaps.

**Figure 1:**
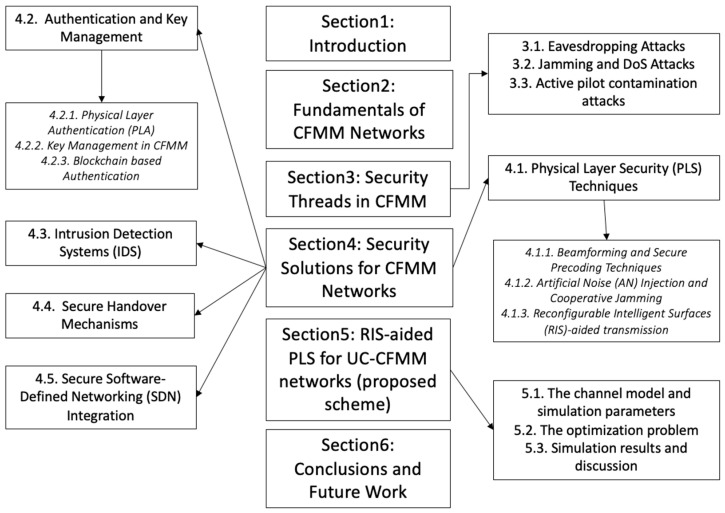
The Paper Outline.

The rest of the paper is organized as follows: Section 2 introduces the fundamentals of CFMM networks and Section 3 provides the security challenges, including eavesdropping attacks, jamming and DoS attacks, and pilot spoofing and contamination. The state-of-the-art techniques proposed to provide security solutions for CFMM systems are detailed in Section 4. The case study of the RIS-aided PLS scheme is introduced in Section 5. Finally, the conclusions and the future work are introduced in Section 6. Figure 1 shows the study outline.

## 2. Fundamentals of CFMM Networks

Unlike conventional cellular networks that depend on specific base stations (BSs) or APs to serve users within designated cells, CFMM [27,28] eliminates the concept of “cells.” Instead, it employs multiple distributed low-power APs across a broad geographical area to meet users’ needs. Each AP is equipped with multiple antennas and is connected to a central processing unit (CPU) via high-speed backhaul links [27]. This approach significantly reduces inter-cell interference and improves the overall service quality across the entire coverage area [29], besides its capability to reduce mobile phone interference and enhance the macro-diversity [29] through ubiquitous and distributed APs. The backhaul network must support rapid data exchange to ensure low latency and high throughput. Both the backhaul and fronthaul (the links from users to APs) need to accommodate the high data rates generated by maMIMO systems [27].

**Figure 2:**
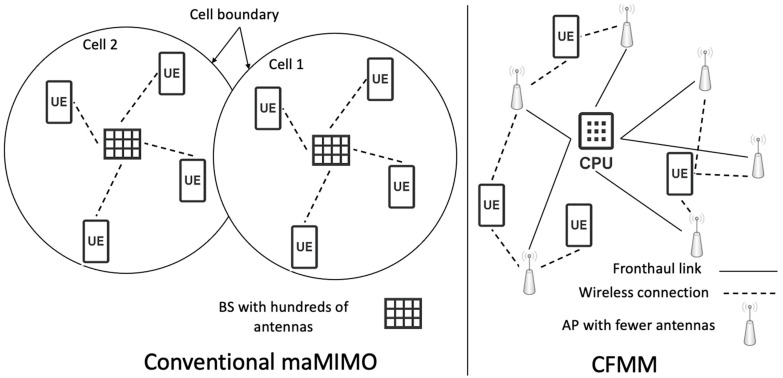
Classic (Conventional MIMO) versus CFMM systems.

Figure 2 shows a classic maMIMO (cellular system) versus a CFMM system [30]. The CPU coordinates all APs in the system. The APs exchange user data and channel state information (CSI) with the CPU, which collaborates to process the incoming signals and optimize transmission for each user. Accurate CSI is crucial for efficient signal processing in CFMM systems. It enables both the APs and the CPU to adjust their beamforming strategies, optimizing data transmission while minimizing both intra-cell and inter-cell interference. Each user in the network is served by a subset of APs chosen based on the user’s location and channel conditions [27].

As users move, the group of APs that serve them changes dynamically, ensuring that they always receive service from the APs with the strongest signals. In coherent processing [31], the APs synchronize their transmissions, allowing signals from multiple APs to combine coherently at the user’s device, thereby increasing signal strength. In contrast, non-coherent processing involves each AP serving users independently. While this method is simpler, it is also less efficient. CFMM offers significant advantages over small-cell systems [32] in terms of maximum rate and power efficiency. To increase the reader’s knowledge, a valuable comparison between cellular, cell-free, and intelligent maMIMO is available in [16].

Cellular networks based on macro cells have served as the foundation of mobile systems since 3GPP Release 8 Long-term evolution (LTE), evolving into 5G NR starting with Release 15. Small-cell networks were introduced in Release 9 with femtocell support and were further developed through LTE-Advanced (HetNets) and feature enhancements in Releases 10 to 12, with continued progress into 5G via Integrated Access and Backhaul (IAB) in Release 16. The 3GPP has not formally defined CF or CFMM as standalone, branded network architectures. Instead, it focuses on standardizing the foundational technologies and functional splits that can support CF-like deployments. Several features introduced in Releases 16 to 18, such as coordinated multi-point (CoMP), multi-TRP (Transmission Reception Point), and distributed Radio Access Network (RAN), contribute toward achieving CF performance characteristics, although no dedicated specification currently exists. A comprehensive table of features organized by Release is also available in the 3GPP Work Plan. Table 2 lists the relevant 3rd Generation Partnership Project (3GPP) standards for each network type.

CFMM faces several implementation challenges, particularly in ultra-dense scenarios. The key aspects of these environments include simplified system design, efficient processing, and flexible resource scalability with low complexity, limitations in fronthaul, handling massive access, maintaining synchronization, and efficient channel acquisition [33]. Scalability is a key challenge for CFMM because deploying numerous APs over a broad area, along with the necessary infrastructure for coordination and backhaul, requires careful planning. Efficient management of resources, such as power, frequency, and AP coordination, is vital for scaling up CF networks while maintaining high performance levels [28].

CFMM relies on users transmitting pilot (or reference) signals to assist APs in estimating communication channels [27,31]. However, when multiple users use the same pilot sequence, it leads to pilot contamination, creating a significant challenge. Pilot contamination and hardware impairments can result in a non-zero estimation error floor. The increasing number of antennas and radio frequency (RF) chains has significantly raised the complexity of symbol detection in the receiver5. Effectively managing these issues and selecting the right UE hardware are crucial for maintaining system performance.

Paper [13] discusses CFMM system models, key performance metrics like energy efficiency (EE) and channel hardening, and outlines current research trends, open issues, and lessons for advancing this technology in wireless communications. Radio stripes (RS) [34] are a sequential fronthaul network used in CFMM systems. They connect APs to a CPU via a single cable, offering advantages over traditional star topologies. Paper [35] provides a comprehensive overview of the principles and recent advancements in CF and RS networks, discussing key processes such as uplink (UL) pilot transmission, channel estimation (CE), and data exchange between APs and user equipment (UE). Table 3 provides a comparison of CFMM security with co-located MIMO and small cell networks.

CFMM is believed to benefit from channel hardening and favorable propagation [31], like cellular MIMO systems. However, these properties are heavily dependent on the propagation environment. Research presented in [41] indicates that channel hardening is generally weak, although deploying multiple antennas per AP can enhance it. Favorable propagation is more likely to occur with smaller path loss exponents, higher antenna density, and spatially separated users. The main takeaway is that channel hardening and favorable propagation should not be relied upon for calculating achievable rates. Instead, CFMM designs should incorporate achievable throughput expressions and resource management schemes that account for impairments such as spatial correlation, pilot contamination, and estimation errors. Achievable rate bounds can be derived using low-complexity linear processing methods [42]. Furthermore, optimal pilot design [43] is crucial; signal detection techniques, including maximum ratio (MR), zero forcing (ZF), and minimum mean square error (MMSE), must be carefully selected to achieve a balance between performance and complexity [5]. In addition to these challenges, the next section will discuss the security issues faced in CFMM networks.

## 3. Security Threats in CFMM Networks

A standard security system is designed to ensure data integrity, confidentiality, availability, and authentication while protecting against both passive and active attackers [44]. The distributed architecture of the CFMM system offers distinct advantages in terms of secrecy compared to traditional cellular networks. Because APs are geographically dispersed, they create highly unique and uncorrelated wireless channels for different users and potential eavesdroppers. Increasing the number of APs enhances the secrecy rate by providing greater spatial diversity and enabling better alignment of signal energy toward legitimate users while creating interference for eavesdroppers. This spatial diversity makes it significantly harder for eavesdroppers to capture useful information, thereby increasing the system’s secrecy capacity (SC) [11]. However, the distributed architecture of CFMM also introduces certain vulnerabilities. For instance, if an AP is physically compromised, an attacker could potentially take control of the network and access sensitive information. Additionally, APs are often deployed in uncontrolled environments [45], such as rooftops or street furniture, which raises the risk of physical tampering. The lack of a central base station to manage the APs adds complexity to network management and security. The distributed APs must work together to coordinate without creating security gaps that attackers could exploit. The most significant challenges facing CFMM networks include eavesdropping, jamming, denial-of-service (DoS) attacks, and pilot contamination.

### 3.1. Eavesdropping Attacks

Eavesdropping, whether passive or active [46], is a significant concern in wireless networks since it involves unauthorized parties intercepting communications between legitimate users [47]. The absence of central control in CFMM makes it particularly difficult to secure all communication links. Furthermore, with APs deployed in proximity, an eavesdropper can easily position itself nearby to exploit vulnerabilities at the physical layer. Sophisticated techniques for eavesdropping, especially passive attacks, pose substantial threats to data confidentiality. These methods allow attackers to listen in on communications without interference. On the other hand, active eavesdropping occurs when an eavesdropper not only attempts to intercept transmissions but also actively transmits signals to disrupt or manipulate communication. This presents a more significant security threat compared to passive eavesdropping [48].

### 3.2. Jamming and DoS Attacks

Malicious entities often attempt to disrupt communication channels through jamming or spoofing. Jamming [49,50] and DoS attacks [22] aim to overwhelm the network with excessive noise or traffic, resulting in service disruption and reducing the reliability and availability of the network. These types of attacks pose significant risks to CFMM networks due to the numerous APs involved and the distributed nature of the system. In jamming attacks, an adversary transmits strong signals that interfere with legitimate user communications, rendering the network unusable. For instance, a jammer may try to disrupt estimating the channel by sending jamming signals during the pilot transmission phase, introducing what is known as pilot contamination [51].

CFMM networks rely on coordination between multiple APs, so the compromise of even a single AP can severely degrade overall network performance and service quality. Furthermore, the decentralized structure of CFMM systems complicates the detection and mitigation of jamming attacks. The spatial diversity in maMIMO technology makes it more challenging for a jammer to simultaneously disrupt communication between all APs and their users. Because the APs are spread over a wide area, a localized jamming attack may only affect a small subset of APs, while others continue to function normally. To effectively disrupt communication, a jammer would need to target multiple locations at the same time. Paper [49] presents a thorough survey of jamming attacks and anti-jamming strategies in various wireless networks.

### 3.3. Active Pilot Contamination Attacks

One reason for pilot contamination is that attackers can transmit signals during the pilot phase, deceiving APs and causing them to inaccurately estimate the CSI. This leads to degraded communication quality, reduced spectral efficiency, and potential eavesdropping opportunities. Pilot contamination is particularly problematic in CFMM systems due to the lack of coordination among APs, which increases the likelihood of attackers injecting false pilot signals into multiple APs simultaneously [52]. Both pilot contamination and active pilot attacks significantly undermine the secrecy performance of maMIMO systems [53]. The literature contains extensive research on pilot contamination attacks [11,54], pilot spoofing attacks (PSA) [55,56], and optimal pilot strategies [43].

Pilot contamination is a threat during both the training and data transmission phases. Estimation errors in high-dimensional channels can result in inaccurate detection/beamforming during transmission and reception, potentially leading to the leakage of confidential messages to eavesdroppers and compromising the system’s security [57]. To detect spoofing attacks, special tests are required, such as using channel virtual representation with Neyman-Pearson testing in static environments and machine learning-based schemes in dynamic environments [58]. Table 4 highlights the differences between the three attack types. The next section explores different facets of security in cell-free (CF) networks to ensure secure and reliable communication in next-generation wireless networks.

## 4. Security Solutions for CFMM Networks

Addressing the security challenges in CFMM networks requires a combination of techniques from both the physical layer and higher network layers. Key countermeasures include, in addition to PLS, distributed authentication, trust management, robust jamming detection and mitigation, and advanced pilot contamination mitigation. Additionally, mechanisms such as hybrid relay-reflecting intelligent surfaces (HR-RIS) [59] are vital for securing APs against physical tampering and unauthorized access. These techniques help ensure the integrity of APs in untrusted environments. The main challenges in these networks arise from conflicting objectives: maximizing information transfer (data rate), providing sufficient power transfer for energy harvesting by legitimate users, and mitigating the impact of active eavesdroppers48. This section provides insights into various aspects of CF security, covering everything from physical layer security to energy efficiency optimization and solutions for addressing active eavesdropping and other security challenges.

### 4.1. Physical Layer Security (PLS) Techniques

Network-layer cryptography faces challenges in dynamic networks, leading to increased interest in PLS30, which offers the potential for perfect security without the complexities of traditional encryption methods [44]. PLS [60,61,62,63] refers to a set of techniques and strategies used to secure communication at the physical layer of the Open Systems Interconnection (OSI) model. This layer, being the lowest in the OSI model, is responsible for the actual transmission and reception of raw bit streams over physical mediums such as cables or wireless channels. PLS aims to protect information from eavesdroppers and unauthorized users by utilizing the unique characteristics of the physical medium. Additionally, PLS has lower computational and communication overhead compared to conventional cryptographic methods, and it can be integrated with higher layers to provide a multi-layered security approach for improved quality of security (QoSec) [64]. PLS leverages the unique properties of wireless channels such as fading, noise, and interference to enhance security at the physical layer in dynamic environments [65]. For instance, CFMM can exploit the inherent randomness present in wireless channels to generate unique keys that are difficult for attackers to guess. Since the channel response between a user and an AP is reciprocal (the same in both directions), this characteristic can be utilized to generate a shared secret key. One significant advantage of PLS is its ability to reduce computational costs and resource consumption [66].

PLS techniques are classified based on various criteria, including fading models, types of eavesdroppers, diversity, and specific applications [65]. For 6G technologies [67], PLS includes channel-based approaches, power allocation strategies, and signal processing methods. Channel-based approaches [60] utilize the characteristics of communication channels for various purposes, including secret key generation and distribution, adaptive coding and modulation, physical layer authentication, dynamic power control, and channel adaptation. They also employ techniques such as artificial fading and channel randomization. These approaches offer real-time adaptability without requiring significant computational power or excessive overhead [65]. On the other hand, power allocation based PLS strategies [68,69] focus on minimizing power consumption while enhancing energy efficiency and ensuring security requirements are met [55]. These power allocation methods, which demonstrate increased resilience to eavesdropping, can be used in conjunction with techniques like artificial noise (AN), MIMO systems, beamforming, cooperative jamming, relay networks, energy harvesting, and rate allocation schemes [2,70].

PLS, which utilizes signal processing [71], is a promising solution for securing wireless communications, particularly in resource-constrained or latency-sensitive environments. As wireless networks advance, these methods are anticipated to play a more significant role in securing next-generation networks. Signal processing based PLS encompasses various techniques, including beamforming and directional transmission, secure precoding, AN injection, channel CSI exploitation, cooperative jamming, secret key generation from physical layer attributes, and frequency hopping and spread spectrum techniques. The authors of [71] presented a comprehensive summary of existing surveys on PLS schemes and defined important secrecy performance metrics such as SC, secrecy outage probability (SOP), and secure energy efficiency (SEE). Book [72] provides an in-depth understanding of the concepts, frameworks, and methods of PLS, as well as its implementation in 5G and other evolving wireless networks. The following subsections discuss key PLS techniques applicable in CFMM networks.

#### 4.1.1. Beamforming and Secure Precoding Techniques

Beamforming [73,74,75,76], a technique that directs the transmission and reception of signals toward specific directions by regulating the phase or amplitude of the original signal. The main categories of beamforming structures are analog (or radio frequency, RF) beamforming, digital beamforming (also known as digital precoding), and hybrid beamforming architectures. While analog beamforming is simple, it is not used in maMIMO due to its performance limitations. Digital beamforming offers better performance than the analog approach but comes with increased complexity. Hybrid beamforming [77] combines the advantages of analog and digital beamforming, making it a common choice in maMIMO systems [78]. Hybrid beamforming emerges as a practical solution for balancing complexity and performance. Secure and adaptive beamforming strategies further fortify the system against eavesdropping and jamming, especially when combined with intelligent antenna selection and power control. Precoding techniques in maMIMO systems, whether linear or nonlinear, enable multi-antenna systems to transmit multiple data streams simultaneously [79]. These techniques involve shaping the transmitted signal based on the known CSI to enhance signal reception at the intended receiver while disrupting it for potential eavesdroppers [73]. Paper [73] provides a comprehensive survey of precoding techniques in maMIMO and beyond 5G networks, covering both linear and nonlinear precoding, machine learning-based precoding algorithms, and strategies for peak-to-average power ratio (PAPR) optimization.

Bit error rate (BER) for real-time implementation is reduced by combining the data detection scheme with various iterative algorithms, achieving near-optimal performance with fewer iterations and significantly reduced complexity across different CSI conditions and modulation schemes [75]. By concentrating transmission energy in specific directions, beamforming not only reduces the likelihood of eavesdropping and jamming attacks but also maximizes the probability of successful signal detection [80]. Implementing secure beamforming techniques requires consideration of the distributed nature of CFMM and the coordination between APs to maintain strong performance in adaptive antenna selection (AAS) [81,82]. Dynamic selection of antennas based on channel quality can help mitigate the effects of jamming. Additionally, beam selection and management using AI [62]. reduces the overhead and complexity associated with beamforming techniques.

Additionally, pilot decontamination strategies are characterized by four key elements: pilot design, pilot allocation strategies, channel estimation techniques, and precoding methods [83]. The joint design of beamforming and user-centric (UC) clustering with multiple cooperating CPUs demonstrates significant performance improvements over traditional architectures, under-scoring the advantages of multi-CPU cooperation in CFMM systems [84]. Conjugate beamforming combined with precoding normalization [85] helps eliminate the need for downlink pilot signals by bridging the performance gap typically associated with conventional conjugate beamforming without training.

Additionally, several studies [86,87,88] present robust approaches to enhance the performance of CF integrated sensing and communication (ISAC) MIMO systems, where distributed APs collaborate in both communication and target sensing tasks. Joint sensing and communication (JSC) beamforming, when integrated with power allocation strategies, can achieve near-optimal SINR for communication and SNR for sensing. However, limitations exist in these studies: study [86] assumes perfect CSI, neglects clutter and multipath effects in sensing, and does not address scalability; [87] relies on idealized propagation models without accounting for practical hardware constraints such as fronthaul limitations; and [88] focuses solely on monostatic sensing with co-located APs, omitting bistatic or multi-static scenarios. Moreover, study [89] leverages machine learning (ML) and deep learning (DL) to dynamically adapt beamforming in evolving ISAC environments, thereby reducing computational overhead. Nevertheless, this approach suffers from challenges, including data dependency, poor generalization to unseen scenarios, and high training complexity.

As a summary, beamforming and secure precoding significantly enhance security and performance in CFMM systems, but their real-world implementation is challenged by several practical issues. These include high computational complexity, real-time and scalability constraints, coordination overhead among distributed APs, and imperfections in CSI due to pilot contamination.

AI-based methods (e.g., DL and graph neural networks (GNNs)) help reduce signaling overhead but are limited by the need for quality training data, generalization ability, and high processing power. Distributed learning can also suffer from inconsistent performance due to local data limitations. Although hybrid beamforming cuts hardware costs, it demands precise analog-digital coordination. Power allocation strategies face trade-offs between performance and energy limits. Overall, while promising in theory, practical deployment requires more adaptive, efficient, and lightweight solutions to overcome these barriers. Table 5 provides a summary of works related to beamforming and secure precoding in CFMM networks.

#### 4.1.2. Artificial Noise (AN) Injection and Cooperative Jamming

AN injection, as described in references [46,60], is a technique in which noise is intentionally added to a transmission to confound eavesdroppers while allowing legitimate users to decode the intended message. In the CFMM system, artificial noise can be distributed across multiple APs, which further complicates the process for eavesdroppers attempting to filter out the noise and intercept the communication. By carefully optimizing the power and direction of the noise, legitimate users can maintain a favorable signal-to-noise ratio (SNR), while potential eavesdroppers face a lower SNR, making it more difficult for them to decode the message. The power control method enhanced with AN introduced in [98] boosts the lowest achievable secrecy rate across users and greatly enhances security in CFMM systems facing passive eavesdropping threats. In related work [100], AN is used to shrink the region around users where eavesdropping might occur. A secure beamforming method is introduced to guarantee a minimum SINR for legitimate users while reducing the eavesdropper’s SINR. The idea of cooperative AN is introduced in [101], where all APs spread the AN signal in the null space of the users’ channel to complicate eavesdroppers’ operation without complex beamforming. As a modification of this approach, the APs are divided into two groups: one for transmitting AN and the other for serving the legitimate users, ensuring perfect incorporation into real-world conditions. In CFMM, the APs can cooperate to detect and cancel jamming signals.

For instance, if one AP detects interference, it can share this information with other APs in the network, which can then take corrective measures to avoid disruption. If one AP detects abnormal interference patterns, it can alert other APs in the vicinity to adjust their transmission strategies accordingly. Cooperative jamming [65] uses friendly nodes in the network to actively interfere with potential eavesdroppers. These cooperative nodes transmit interference signals that degrade the eavesdropper’s channel, making it more difficult for them to intercept the communication. Legitimate users can still receive and decode the information because they have a higher SNR relative to the eavesdropper. The system PLS defines two roles of cooperative nodes in the network: the first role is cooperative relaying, and the other is working as a cooperative jammer. Traditional AN can degrade the legitimate channel’s capacity and poses challenges in selecting optimal designs. To overcome these issues, as an alternative recent study [102] introduced a non-orthogonal AN scheme using a pilot information codebook. This approach allows the receiver to identify and cancel the transmitted non-orthogonal AN signal.

Based on the channel state. It boosts secrecy capacity without adding noise to the legitimate channel (without affecting BER), as confirmed by theoretical analysis and simulation results. Table 6 discusses the contributions and limitations of works related to AN injection scheme in CFMM networks.

#### 4.1.3. Reconfigurable Intelligent Surfaces (RIS)-Aided Transmission

The research community has transitioned its focus toward post-5G network architectures, with significant emphasis on physical layer advancements for 6G. Among the most promising technologies are: (1) CFMM systems and (2) intelligent reflecting surfaces (IRS), also referred to as Reconfigurable Intelligent Surfaces (RIS) [106]. These paradigms have emerged as leading physical layer candidates for 6G communication systems and are concurrently regarded as strong enablers for PLS in next-generation wireless networks [38]. RIS [107,108] is recommended for CFMM networks to address the challenges of harsh propagation conditions by maximizing the total user rate through optimally designed beamforming techniques.

**Figure 3:**
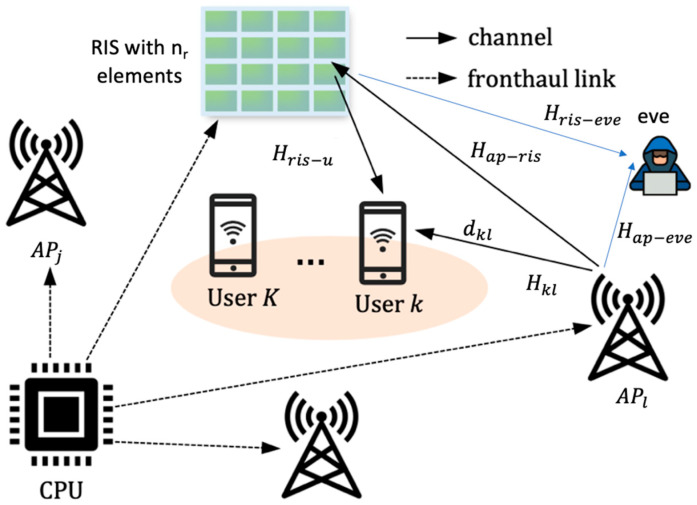
Conceptual diagram of RIS-aided CFMM propagation.

The passive nature of RIS technology makes it resilient to receiver noise, facilitates easy deployment, and requires less complex signal processing [71]. Figure 3 illustrates the conceptual diagram of RIS-aided CFMM propagation. RIS-aided CF networks [109] offer several advantages: they enhance signal strength and coverage, improve network throughput and latency, allow for dynamic network adjustments, reduce costs and power consumption, and ultimately increase network security [9].

Combining AN with multiple RISs improves the secrecy rate by finding optimal AP beamforming and joint optimization of RIS phase shift and AN matrix [37,71]. The implementation of IRS effectively enhances the achievable lower bound in the presence of pilot contamination attacks. Furthermore, an increased correlation coefficient between the legitimate and eavesdropping channels does not uniformly degrade the lower bound. Conversely, the secret key rate exhibits a decreasing trend with the escalation of Eve’s transmit power [110].

Table 7 summarizes the work related to this subsection. As indicated in Table 6, efficient implementation of RIS-assisted CFMM for security requires accurate CSI, intelligent beamforming, robust optimization, and scalable deployment strategies. Scientific research directions should focus on practical hardware constraints, real-time adaptability, and AI-driven security enhancements. Showing clear performance gains when combining IRS-aided passive beamforming with AN [111]. However, practical deployment challenges remain, especially regarding incomplete CSI, algorithm scalability, hardware non-idealities, and transmit power trade-offs.

### 4.2. Authentication and Key Management

Authentication techniques [118] play a crucial role in MIMO systems by verifying the identity of users, devices, and network components. In traditional cellular systems, authentication typically occurs between users and a central BS. However, in CFMM networks, multiple distributed APs collaborate without a central control node. This decentralized architecture adds complexity to the authentication process, necessitating more advanced techniques to ensure security while maintaining system efficiency. By leveraging spatial diversity, channel reciprocity, and decentralized architectures, CFMM can provide robust security against evolving wireless threats. The findings in [118] highlight the dependence of authentication performance on the antenna array size, demonstrating that the mutual information-based authentication rate increases as the number of antennas grows. UEs must authenticate themselves to the network to confirm its legitimacy, typically using public-key infrastructure (PKI) or certificate-based systems. Additionally, the network must authenticate itself to the user to prevent security breaches, as malicious APs may attempt to impersonate legitimate ones. Authentication methods are categorized into three main types: traditional cryptographic methods including symmetric and asymmetric cryptography [119,120], physical layer authentication (PLA) [121,122,123] and emerging technologies such as blockchain based authentication [45,124]. In distributed systems like CFMM networks, group-based authentication can be implemented, where a cluster of APs collaborates to authenticate a user, sharing resources and information to enhance security and reliability [125,126]. Due to the large number of antennas and the decentralized architecture of CFMM [127,128], it is essential to guarantee that only authorized devices can access the network and that cryptographic keys are exchanged securely.

#### 4.2.1. Physical Layer Authentication (PLA)

New security methods have emerged at the physical layer, offering lightweight alternatives to traditional techniques. PLA includes three typical architectures [121]: authentication leveraging CSI, device-specific RF features, and identity watermarking. It is observed that channel-based PLA exhibits higher sensitivity to variations in the communication environment and device mobility. In contrast, RF fingerprinting (RFF)-based approaches [122] encounter challenges related to robust feature extraction and classification accuracy in complex network scenarios. Watermark-based PLA shares a structural similarity with traditional cryptographic techniques such as digital signatures, but its security is grounded in key equivocation principles.

Physical layer information from the wireless channel can be captured using metrics like CSI, Received Signal Strength (RSS), channel impulse response (CIR), or signal phase [129]. These characteristics are intrinsically linked to both the wireless environment and the hardware of the device, which makes them stable and resistant to user manipulation. Consequently, they can be reliably used to identify wireless transmitters [123]. The survey [122] offers a comprehensive summary of PLS methods for ensuring confidentiality, authentication, and malicious node detection. It introduces a structured taxonomy that further classifies each of these categories. the paper reviews recent advancements in channel-based, device-based, and tag-based PLA, and examines how PLA integrates with emerging physical layer technologies.

CSI-based approaches [130] rely on the exceptional characteristics of the physical communication channel, which vary over time and space. By using the shared wireless channel as a source of randomness, legitimate users can create identical keys that are challenging for eavesdroppers to predict or replicate. The concept of channel reciprocity [131] refers to the phenomenon where the channel characteristics between two communicating parties are the same in both directions of transmission, uplink and downlink. In CFMM, this property can be leveraged to generate secret keys for encryption without relying on higher-layer cryptographic algorithms [132]. The reciprocity property enables legitimate users to observe the same impulse response within the channel’s coherence time, whereas the inherent variability of the wireless channel has a direct impact on the achievable generating key rate [133]. If legitimate users and an eavesdropper experience different channel condition, signal processing techniques [134] are utilized to adapt the transmission in a way that enhances security.

By employing dynamic and adaptive coding schemes, legitimate users take advantage of the distinct channel conditions to gain an edge over the eavesdropper. With cooperative transmit power allocation at APs the achievable SC is maximized. Physical layer attributes [60,135] such as channel fading, noise, Doppler shifts, the radio channel entropy [136], and channel correlation characteristics [137] can serve to generate shared secret keys between legitimate users, which are unique to the specific communication channel and difficult for eavesdroppers to predict. Techniques such as channel quantization and randomness extraction are used to convert the physical attributes into usable cryptographic keys [131]. In maMIMO systems, having many antennas provides a high spatial resolution, which can be effectively used for generating secure keys. As the antenna count grows, sophisticated signal processing schemes like precoding are used to direct the transmitted energy [132]. For confidentiality, both keyless and key-based approaches were outlined, including techniques such as secrecy-capacity codes and AN. In practice, key-less PLA [61] ensures confidentiality by directly encoding data over the communication channel. As a result, system performance heavily relies on knowledge of the channel’s characteristics, like the SNR, and the capabilities of prospective eavesdroppers. However, accurately assessing an attacker’s profile in advance is often challenging. If an eavesdropper turns out to be more powerful than anticipated, such as possessing numerous antennas or a low-noise receiver, the actual security capacity may fall below the intended data rate, rendering the system inherently insecure. In contrast, key-based PLA relies on well-established symmetric encryption methods to ensure confidentiality, concentrating exclusively on generating keys through channel reciprocity-based techniques. This separation enables flexible key renewal on demand or adjustment of key strength when needed. For these reasons, key-based PLA is more suitable for integration into a practical and comprehensive security framework [136].

Paper [129] proposes an efficient physical layer key generation technique that utilizes the RSS of wireless signals to establish shared keys between communicating parties, based on the channel reciprocity principle. The authentication mechanism’s capability to dynamically switch between CSI-based and cryptographic authentication, contingent on the real-time CSI condition, enhances its adaptability to environments with intermittent or periodic mobility. By continuously monitoring the CSI state, the system can also detect unauthorized physical perturbations or mobility of wireless nodes, thereby enabling anomaly detection and triggering alerts for potential security breaches [138]. Location-based authentication and fingerprint embedding in MIMO systems [131] achieve strong security even with imperfect CSI. Paper [139] examines the application of AN in fingerprint embedded authentication under the condition of imperfect CSI at both the transmitter and receiver. While AN improves security, its effectiveness diminishes as CSI quality worsens. The analysis in [139] highlights operating regimes that optimize AN use, track the adversary’s knowledge of the key, and suggest potential applications for privacy amplification, while noting that increased AN power may disrupt the adversary’s key recovery. Reference [64] discusses the integration of localization-based node authentication with secret key generation (SKG). Additionally, authentication techniques in MIMO systems and adaptive PLA have been studied from an information-theoretic standpoint in [118]. The combination of SKG with physical unclonable function (PUF)-based authentication protocols [140] offers a substantial reduction in both authentication and key generation latency when compared to conventional methods. The proposed authenticated encryption scheme, which incorporates SKG and a resumption protocol, enhances both security and latency performance. Additionally, several studies [123,141,142,143] explore PLA using ML techniques, highlighting their potential in improving wireless communication security [144]. The concept of multifactor authentication (MFA) [145,146,147] is also relevant here, as it strengthens security by combining multiple authentication factors—such as knowledge (something you know), possession (something you have), and inherence (something you are).

#### 4.2.2. Key Management in CFMM

In the communication process, pilot signals used for channel estimation can act as a source of randomness for generating cryptographic keys. Both the UE and APs observe the received pilot signals and extract secret keys from these observations. Furthermore, as noted earlier, the inherent wireless channels noise and interference can enhance the randomness required to generate shared secret keys between UEs and APs [148]. Once authentication is established, whether by traditional cryptographic methods or by key-based PLA, the next step is to securely manage cryptographic keys [20]. A proper key management process includes four operations: key analysis, key assignment, key generation, and key distribution. To achieve this, public-key cryptography methods such as RSA [149,150] or Elliptic Curve Cryptography (ECC) enable the user and APs to securely establish shared secret keys over an insecure channel. This is accomplished using key exchange protocols like Diffie-Hellman (DH) or Elliptic Curve Diffie-Hellman (ECDH) [45], which allow two parties to generate a common shared key. In some scenarios, pre-shared keys may be utilized between the UE and the APs. While this method is less flexible and scalable, it can provide high security in closed environments. For highly secure systems, quantum key distribution [148,151] might be employed, taking advantage of quantum communication channels to exchange encryption keys in a manner that is fundamentally secure against eavesdropping. To ensure continued security, keys must be periodically refreshed. This prevents the reuse of compromised keys and enhances overall security.

In CFMM, key refresh mechanisms need to be synchronized across distributed APs to ensure seamless communication. If a user or AP is compromised, key revocation protocols must be in place to ensure that compromised keys can no longer be used. Revocation lists or blockchain-based methods can track and invalidate keys across the system. Distributed APs must be synchronized in their key management operations to prevent inconsistencies, which could lead to dropped connections or vulnerabilities [124]. Lightweight cryptographic protocols are premeditated to be computationally efficient while still providing strong security. These protocols reduce the processing load on APs and UEs while ensuring secure key exchanges and data encryption. Ensuring scalable key management without overloading the system is a challenge. Key exchange and management protocols must be efficient to avoid introducing excessive latency, which can degrade the performance of real-time applications. paper [152] examined a range of key management protocols designed for group communication in both wired and wireless networks. the paper analysis focused on identifying security vulnerabilities, evaluating the advantages and disadvantages of each protocol, and comparing their performance with related methods. However, due to the diverse requirements of different applications and environments, no single solution can address all challenges. Therefore, the most appropriate protocol should be selected based on the specific needs of each application.

#### 4.2.3. Blockchain-Based Authentication

As a decentralized system, blockchain [45,124,147,153] can be utilized to ensure secure and transparent authentication of devices. In this context, APs can function as nodes within the blockchain, maintaining a secure ledger of user identities and their access rights. Blockchain is a decentralized, distributed digital ledger or database that records transactions across multiple computers in a way that prevents retroactive alteration of the recorded transactions. Each “block” in the blockchain contains a list of transactions [15], and these blocks are sequentially linked, forming an unbroken “chain”. Key advantages of blockchain technology include decentralization, immutability, transparency, and security [124,153]. It enables decentralized access control, ensuring that only authorized devices are permitted to connect to the network. Additionally, blockchain facilitates secure data sharing among different nodes in the network, maintaining data integrity and authenticity. With its numerous security benefits, blockchain is becoming an increasingly popular tool for enhancing data protection, identity management, and secure transactions [147].

Blockchain technology distributes data across multiple nodes, which contributes to its decentralized nature. This decentralization significantly reduces the risk of a single point of failure and makes it more challenging for attackers to compromise the entire network. Since there is no central server to target, blockchain-based systems are generally more resilient to DDoS attacks. Once data is recorded on a blockchain, it becomes immutable. This means that it cannot be altered or deleted without the consensus of the network, ensuring that the history of transactions is permanent and resistant to tampering. Blockchain provides a transparent and verifiable record of all transactions, which creates a clear audit trail. This feature is particularly beneficial in fields such as supply chain management and financial transactions, where accountability is essential [154].

Moreover, blockchain employs cryptographic techniques like public–private key pairs and hashing algorithms to secure transactions and verify the identities of participants. As a result, it becomes extremely difficult for unauthorized parties to alter or manipulate data. Blockchain can also be utilized for secure identity verification and management without relying on centralized intermediaries, thus reducing the risk of identity theft [124]. It can secure communications and data sharing while preventing unauthorized access to systems. By tracking the provenance of goods at every stage, blockchain ensures their authenticity and integrity. Additionally, it enhances the security of digital payments and banking through immutable transaction records. Blockchain also facilitates the creation of decentralized, self-sovereign identities, reducing reliance on centralized identity providers. By leveraging these features, systems can significantly improve their overall security, making it more difficult for attackers to compromise them and ensuring higher levels of data integrity and privacy. The integration of blockchain with CFMM networks is an intriguing area of research, particularly regarding decentralizing network management, enhancing security, and enabling new business models. However, significant technical, scalability, and regulatory challenges must be addressed before this integration can be implemented effectively on a large scale. Papers [155,156,157,158] provide appreciated insights into the incorporation of blockchain in next-generation networks. Table 8 provides scientific research papers interrelated to Authentication and Key Management in the CFMM system.

### 4.3. Intrusion Detection Systems (IDS)

IDS is a security technology used to detect and respond to malicious activities or violations within a network. Its primary role is to monitor network traffic, identify suspicious behavior, and alert network administrators to potential threats [47]. In CFMM networks, IDS are essential for identifying and mitigating unauthorized access, cyberattacks, and other security threats [134]. These networks lack fixed cell boundaries, increasing security risks due to open access to multiple users. IDS techniques for CFMM include machine learning-based IDS, distributed IDS, and centralized-distributed coordination. Machine learning-based IDS detects anomalies by analyzing large datasets from multiple APs, leveraging models that can learn the normal behavior of users and detect deviations and unusual patterns in network traffic. Distributed IDS deploys IDS agents across multiple APs, enabling localized detection and response. Distributed IDS can provide real-time threat detection and response. Centralized-distributed coordination IDS uses a hybrid architecture where distributed IDS nodes collect data and report to a central controller, balancing scalability, and response time.

As examined in [160], to detect unauthorized access during the authentication phase, structured datasets are created using statistical features derived from signals received at the AP. These datasets are used to train support vector machine (SVM) classifiers. Artificial training data is generated by simulating the transmission and feature extraction processes at the AP. Two types of SVM classifiers are employed: a twin-class SVM (TC-SVM), suitable when full CSI is available, and a single-class SVM (SC-SVM), more appropriate when only legitimate users’ CSI is known. The effectiveness of these classifiers is assessed based on kernel function selection, feature choice, and the eavesdropper’s transmission power. Results show that achieving over 95Performance metrics, such as outage probability, are useful for detecting pilot attacks, as demonstrated in [161]. Jamming or Sybil attack detection introduced in [122] using either single or multi-parameter strategies. the two categories: Jamming-Attack Detection and Composite Jamming-Attack Detection schemes both relies on the difference in noise variances between two nodes. Signal anomalies, as discussed in [47], can be effectively identified using log-likelihood ratio tests implemented in both centralized and decentralized systems, achieving strong detection performance without notably impacting the SE of data transmission. Randomizing the pilot sequence exchanged between Alice and Bob has also proven to be an effective defense against injection attacks. On the other hand, reactive jamming presents a more advanced threat. In this scenario, the jammer passively monitors the spectrum and activates jamming only when an ongoing transmission is detected. Due to their covert nature and low likelihood of detection, reactive jamming attacks pose a serious challenge to secure and reliable communication [133].

In [162], the authors introduce a lightweight, real-time detector to observe and control performance metrics that are affected by the attacked network in both centralized and distributed modes. The study [163] provides meaningful insights into improving communication security through PLA techniques based on wireless fingerprinting. The fingerprinting differentiates legitimate nodes from potential attackers by utilizing the exceptional characteristics of the wireless channel. To support this approach, various ML techniques for anomaly detection are applied, incorporating a broad set of channel attributes under time-varying conditions. Specifically, four one-class ML strategies are examined and compared: decision-tree-based, kernel-based, clustering-based, and nearest-neighbor-based methods. The results indicate that utilizing multiple channel attributes significantly enhances detection accuracy. Notably, the kernel-based approach delivers the highest accuracy, while the nearest neighbor method offers comparable performance with much lower complexity and no training requirements, making it particularly well-suited for dynamic environments. The remaining two approaches demonstrate slightly lower accuracy but benefit from reduced computational demands.

Current smart grid systems [22] are vulnerable due to manual network management and hardware/software anomalies, leading to the adoption of SD-SGs for automated monitoring, control, and improved security. A supervised learning-based detection scheme for RF jamming attacks in Vehicular Ad hoc Networks employs k-nearest neighbors and random forest algorithms, utilizing features such as the variation in relative speed to effectively distinguish DoS jamming attacks from interference. Additionally, Paper [50] introduces a proactive approach to differentiate RF jamming from interference, emphasizing the significance of relative speed variations and other physical-layer metrics in detecting malicious jamming and enhancing prediction accuracy. Table 9 indicates IDS in CFMM networks.

### 4.4. Secure Handover Mechanisms

While scalable CFMM performs well under static conditions, its dynamics related to AP handover in mobile networks have not been fully explored [166]. Handover refers to the process of selecting a suitable AP to serve a UE during mobility. This process involves optimizing user association decisions while managing computational complexity [167]. The unique characteristics of quantum channels between APs and UEs can facilitate secure location tracking and positioning. This capability can assist with resource allocation and mobility management, ensuring that APs effectively serve the correct UEs, even in densely populated environments. Handover in CFMM systems poses significant challenges [24] due to the high density of distributed APs and the need for precise coordination. Effective handover necessitates intelligent AP selection that accounts for network topology, load distribution, signal strength, and interference levels. Achieving seamless transitions also depends on the synchronization of beamforming vectors between UE and APs, that enforces the need for the cooperative beamforming schemes. Interference from adjacent APs further complicates handover, requiring integrated strategies for beamforming coordination and interference suppression. Employing soft handover techniques [168,169] that enable overlapping coverage areas can minimize communication disruptions and improve reliability. Additionally, adaptively adjusting AP clusters based on UE location and mobility facilitates more responsive and efficient handover decisions [170].

ML approaches offer further enhancement by predicting UE mobility patterns, optimizing AP selection, and improving the coordination between beamforming and handover management [171]. Paper [172] explores the secrecy performance of CFMM networks under the effect of active pilot spoofing attacks by multiple Eves. To mitigate the impact of such attacks, a joint AP selection and power control framework aimed at enhancing the PLS of the system. The design aims to augment the sum SE of the legitimate users while ensuring strictly positive secrecy SE for all users compromised by spoofing attacks. Additionally, in support of practical system deployment, two complementary procedures introduced: (1) a detection scheme for identifying the presence of multiple active Eves and associating each with its corresponding targeted user, and (2) an estimation technique for inferring the large-scale fading coefficients between the APs and the Eves based on received pilot statistics [172].

Authentication during handover, along with fast re-authentication techniques [173], helps to prevent attacks and reduces the vulnerability window during the transition from one AP to another. In CFMM networks, the serving cluster of APs changes dynamically as UEs move. One study [174] provides an inclusive analysis of 50 research papers focused on handover approaches in 5G heterogeneous networks, emphasizing their critical role in ensuring seamless connectivity for user equipment. Additionally, another review [175] examines recent handover decision algorithms, focusing on procedural aspects, performance impacts, self-optimization, and associated challenges, while categorizing algorithms and discussing evaluation methods. While SE performance is enhanced with secure handover techniques, there is no discussion on the signaling overhead or computational cost of handovers in scalable deployments. Table 10 describes the contributions and limitations of some related work for this section.

### 4.5. Secure Software-Defined Networking (SDN) Integration

Integration of SDN in CFMM Networks is a key innovation to improve the flexibility, scalability, and network security. SDN was developed to streamline network administration and computerize infrastructure distribution in wired and wireless networks [162]. SDN allows centralized control of network resources by decoupling the data plane that handles traffic from the control plane, which makes decisions about traffic [15].

When integrated with CFMM, SDN helps optimize resource management, enable dynamic network adaptation, and enhance network security by dynamically adjust security policies based on immediate network conditions. SDN supports in identifying and isolating malicious traffic, preventing it from spreading across the network [178]. In CFMM networks, the CPU acts as an SDN controller that redirects traffic away from congested or under attack areas of the network, balancing the load across other APs or network segments, and ensuring the optimal coordination between APs [179]. Dynamic and flexible mapping between CPUs and APs enables the real-time reallocation of APs from congested CPUs to underutilized ones. This dynamic reassignment mitigates fronthaul bottlenecks by balancing the computational and communication load across the network. Moreover, such adaptability enhances system robustness, allowing for seamless handling of CPU failure events through automated AP migration mechanisms.

SDN also facilitates network programmability by enabling CPUs to utilize standardized SDN southbound protocols to issue control-plane directives such as configuration commands, status updates, and event notifications to the APs in a scalable and consistent manner. Although distributed SDN has been thoroughly investigated within the domain of traditional computer networks, its application to CF wireless systems remains largely unaddressed. This gap presents a compelling direction for future research, particularly in the context of scalable and resilient next-generation radio access networks (RANs) [178]. While there are few papers specifically combining secure SDN control with CFMM, several works provide architectural and security frameworks relevant to SDN-driven networks. The following works are closely related and offer valuable context as summarized in Table 10.

The SDN controller dynamically coordinates PLS techniques across multiple APs to ensure that key generation processes are synchronized across APs, improving the reliability of the keys generated through wireless channels. SDN dynamically configures beamforming strategies across APs to avoid eavesdroppers or jammers, ensuring that communication remains secure [90]. The southbound interface serves as the communication link between the SDN controller and network devices.

Securing this interface is critical to preventing attacks. OpenFlow [180] is one of the most widely used southbound protocols in SDN. To safeguard OpenFlow communications, encryption methods like TLS (Transport Layer Security) are used. This prevents attackers from intercepting or tampering with control messages exchanged between the controller and the devices. The SDN controller monitors spectrum usage and detects abnormal patterns of interference, which may indicate a jamming attack. If jamming is detected, the controller reconfigures the network dynamically by switching frequencies, adjusting transmission power, or steering beams away from the jammer, minimizing its effect on communication [22]. In multi-controller SDN architectures, the east–west interface facilitates communication between different SDN controllers. It is crucial to encrypt and authenticate this communication to maintain consistency and to protect against attacks that could manipulate control information between the controllers.

SDN enables network slicing, which allows the creation of multiple virtual networks on top of a single physical infrastructure. By integrating ML algorithms with SDN, the controller can detect and respond to anomalies in real time. ML algorithms can be used to detect unusual traffic patterns that indicate potential security threats. Based on real-time network analysis, SDN controllers can adapt security policies to evolving threats, deploying countermeasures dynamically. Blockchain is also used with SDN [180]. SDN enhances the management of the distributed, dynamic nature of CFMM networks, allowing centralized control while offering dynamic, fine-grained security policies. The centralized nature of SDN provides a powerful mechanism for real-time threat detection and mitigation, but it also introduces challenges such as controller security and securing communication across interfaces.

Paper [181] focuses on securing SDN control planes via Graph Convolutional Network (GCN)-based detection and partitioning mechanisms for real-time mitigation of packet injection DoS attacks. By employing strong encryption, distributed security measures, anomaly detection, and real-time traffic control, secure SDN integration can significantly enhance the robustness of CFMM networks, making them more resilient to attacks and ensuring the integrity and confidentiality of wireless communications. Combining CFMM with SDN/NFV (Network Function Virtualization) frameworks could enable dynamic security adaptations (e.g., AP or RIS selection, AN injection policy, beamforming reconfiguration), representing fertile ground for future work [182]. There is minimal literature explicitly integrating SDN/NFV-based controllers into secure CF deployments, despite CFMM being a candidate for open RAN (O-RAN) and virtualization. Additionally, techniques like anomaly detection, trust-based access control, or packet-injection mitigation applied in CF networking remain largely unexplored. Table 11 provides some related papers.

## 5. Case Study: Secure RIS-Aided CFMM Example

This section presents a comprehensive case study evaluating the effectiveness of a joint RIS, AN injection, and RZF precoding framework for PLS enhancement in CFMM networks. The analysis is performed using an extended simulation environment that incorporates cascaded channels, discrete-phase RIS control, alternating optimization BCD, and greedy elementwise RIS updates. Three representative transmission schemes are compared to quantify their relative secrecy performance, robustness, and energy efficiency. In the joint BCD RIS-aided PLS scheme, RIS cycles through a pre-shared pseudo-random sequence of phase profiles, known exclusively to the APs and legitimate UEs. That disrupts pilot contamination attacks. Adapting AN allocation to the instantaneous channel conditions, and joint optimization of AP precoders, AN covariance, and RIS phase shifts provide multi-security levels aim to increase the minimum SR.

### 5.1. Simulation Framework

The system model is as described in Figure 3 with single antenna APs and single eavesdropper. The channels follow the Rayleigh fading channel distribution [104], the small-scale fading channel h∼CN (0, 1). The channel model is described as: $H=h\;\sqrt{\;d^{-\alpha }\;}\;,$ where d is the path length. All simulation parameters and configuration settings are indicated in Table 12.

The simulator models a CFMM system with L = 24 distributed single-antenna APs, K = 3 users, and a passive N_RIS_ = 64-element RIS arranged on a circular layout. Large-scale fading follows distance-based pathloss with different exponents for AP–user, AP–RIS, and RIS–user links. Monte-Carlo (MC) simulations are executed, each with randomly generated user and eavesdropper positions.

All channel components (direct AP–UE links, cascaded AP–RIS–UE links, RIS–eavesdropper links, and AP–eavesdropper links) are synthesized using complex Gaussian fading combined with large-scale attenuation. The RIS applies a diagonal phase-shift matrix. $\theta =\;diag\left(e^{j\theta_{1}},\ldots ,e^{j\theta_{n_{r}}}\right),\;and\;\theta_{n_{r}}$ the phase shist of element $n_{r}$. For passive RIS | $e^{j\theta_{n}}$| = 1. The effective channel with the RIS-aided scheme (far field) is [111]:

(1) $$H_{eff}=H_{kl}+H_{ap-ris}.\theta .H_{ris-u}$$

and similarly, the effective eavesedropper channel is given by:

(2) $$H_{eff-eve}=H_{ap-eve}+H_{ap-ris}.\;\;\theta .H_{ris-eve}$$

The received SINR at user (k) and the eavesdropper are computed following the standard CFMM signal model with interference, AN leakage, and noise.

### 5.2. The Proposed BCD-Based Joint Optimization

The proposed scheme employs two nested optimization loops:

(a) Precoder + AN design Given current RIS phases, the APs compute a ZF-based precoder. ZF beamforming matrix (the precoding vector) [106]:

(3) $$\mathrm{W\_ZF}=H_{eff}{.\left(H_{eff}^{T}.H_{eff}\right)}^{-1}$$

Using equal per-user data power allocation, the power allocation with AN injection at the null space of the signal and with AN fraction = AN_fraction (equal 0.3) is:

P_data = P_total (1 − AN_fraction), and P_AN = P_total (AN_fraction)(4)

(b) Greedy elementwise RIS update

For each RIS element (n), the algorithm tests discrete phase candidates phase $\theta_{n}\;\in \{0,\;2\pi /L,\ldots ,\;2\pi (L-1)/L\}.$ and selects the phase that maximizes the minimum secrecy rate across users.

(c) Alternating BCD process

The two blocks alternate for several iterations:

1. Fix RIS phases and optimize the beamforming with AN.
2. Fix the precoder and AN covariance and optimize RIS phases.
3. Repeat until convergence or reaching maximum number of iterations.

Five schemes are compared: Baseline CF (no RIS, no AN) Standard CF-ZF transmission without PLS enhancements, CF with RIS (fixed phases) RIS used with random but static phase configuration; no AN, CF with AN (no RIS) AN is injected via null space projection but RIS is absent, Proposed RIS with AN with pilot-hopping model Independent RIS configuration used with AN; eavesdropper receives attenuated pilot-based channel estimate, and Joint BCD (proposed final scheme) Full alternating optimization with greedy RIS updates and ZF with AN design.

### 5.3. The Performance Metrics

Four evaluation metrics are computed:

(a) Per-user secrecy rate

The user’s and eve’s SINR are calculated based on (5) and (6), respectively:

(5) $$\;\;SINR_{k}=\frac{{\left|h_{k}^{H}.w_{k}\right|}^{2}}{\sum_{i\;\neq k}^{K}{\left|h_{k}^{H}.w_{i}\right|}^{2}+\;\sum_{m}{\left|h_{k}^{H}.\;\;an_{m}\right|}^{2}+\;\sigma^{2}}$$

(6) $$SINR_{eve}=\frac{\delta .\;\;{\left|\;h_{eve}^{H}.w_{k}\right|}^{2}}{\sum_{m}{\left|h_{eve}^{H}.\;\;an_{m}\right|}^{2}+\sigma^{2}}$$

where

- $h_{k}$ and $w_{k}$ are the k-th user’s channel and precoder, respectively.

- $an_{m}$ is the m-th AN vector.

The secrecy rate as defined in [109] is given by:

(7) $$R_{s}={\left[{log}_{2}\left(1+{SINR}_{k}\right)-{log}_{2}\left(1+{SINR}_{eve}\right)\right]}^{+}$$

(b) Secrecy Outage Probability (SOP):

(8) $$\mathrm{SOP}=\mathrm{prob}(R_{s}<R_{th}),\;R_{th}\;\mathrm{is}\;a\;\mathrm{threshold}\;\mathrm{rate}\;(R_{th}=0.01)$$

This captures worst-case risk of secrecy failure.

(c) Energy Efficiency (EE): A simple linear power model is used:

(9) $$\mathrm{Energy}\;\mathrm{efficiency}EE=\frac{\mathrm{Average secrecy rate}}{\mathrm{total consumption power}}$$

(d) Complexity analysis: Table 13 indicates the complexity of compared scheme.

### 5.4. The Optimization Problem

The greedy element wise RIS optimization is performed over discrete phase candidates and its objective is to maximize the minimum secrecy rate *$R_{s}$*. The optimization problem: max-min SR (fairness):

(10) $$\begin{array}{c} \underset{w_{k},\theta ,Q_{\mathrm{an}}\geq 0}{\max}\;\;\underset{k=1,\;2,\ldots ..K}{\min}{[R}_{s}\;{\;\;\;[R}_{s}(w_{k},\;\;\theta ,\;\;Q_{an})]\\ \mathrm{s.t.}\\ \;\;\mathrm{the}\;\mathrm{power}\;\mathrm{constrains}:\;\sum_{K}{∥w_{k}∥}^{2}+tr\left(Q_{an}\right)\leq \;\mathrm{P\_total}, \end{array}$$

Using BCD, (10) can be divided into two blocks:

(1) The first block optimizes the precoder and AN design with fixed θ, we use the low complexity heuristic method [104,105]. The optimized precoding vector **W_ZF_opt_ = [** $w_{1opt},w_{2opt},\ldots ,w_{Kopt}]$ is selected to meet P_data and to place AN in the null-space of H with **H = [**$h_{1},h_{2},\ldots ,h_{K}]$ and (**H^T^ W_ZF_opt_**) = I (the identity matrix). These ensure near zero interference at legitimate UE.

(2) In the second block, the RIS phase is updated for fixed $w_{k}$ and $Q_{an}$. Now the non convex problem is to optimize θ to solve (10) for a given precoding vector and known AN covariance. In our simulation, we use the element-wise greedy search method as follows:
  - For the n-th RIS element, try a small set of candidate phase {0, 2π/L,…, 2π(L − 1)/L}. Then compute $h_{k}(\theta )$ for each candidate.
  - Pick the more suitable phase that achieve the objective in (10).
  - Sweep across all elements for several passes.

### 5.5. Simulation Results

Based on the equations defined in previous subsections, we evaluated the proposed scheme and its improved version in comparison with the other three schemes. In the training procedure, we used 200 iterations. In addition to the SR, the SOP and the EE are the performance metrics used in our simulation. The SOP and EE are defined in (8) and (9) respectively.

**Figure 4:**
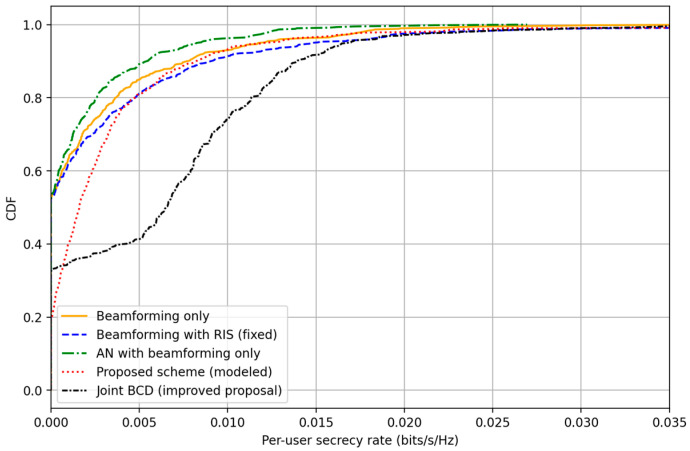
CDF of per-user SR of various secure transmission schemes in a CFMM network (L = 24, K = 3, n_r_ = 64, iterations = 200).

**Figure 5:**
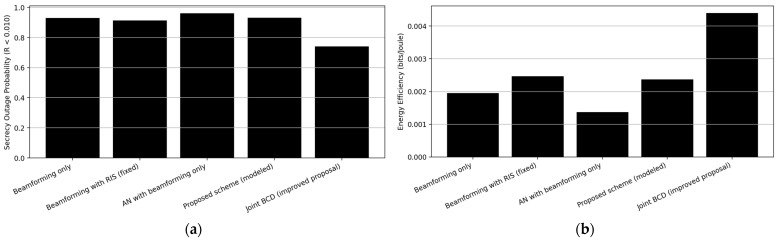
Comparative performance of various secure transmission schemes in a CFMM network: (**a**) The SOP; (**b**) The EE.

Figure 4 and Figure 5a,b illustrate the comparative performance of various secure transmission schemes in terms of SR, SOP, and EE, respectively. Figure 4 shows the CDF of the user’s SR of various secure transmission schemes. As indicated in Figure 4, the joint BCD scheme outperforms other schemes. highlighting the potential of adaptive optimization of the intelligent surfaces to enhance PLS without requiring knowledge of Eve’s channel, followed by the beamforming with the RIS (fixed) scheme due to the additional passive beamforming gain that strengthens the desired signals and weakens the eavesdropping links.

The proposed joint BCD demonstrates the most favorable performance, achieving the highest EE and the lowest SOP among all compared schemes. In contrast, employing AN without RIS leads to a slight degradation in EE (Figure 5b) owing to the increased power consumption for noise generation, while only offering limited secrecy gains when AN is not optimally allocated. AN with beamforming only produces the lowest SR and EE with the highest SOP, as shown in Figure 4, Figure 5b and Figure 5a, respectively. AN degrades the user performance more than it helps jam Eve as it is sensitive to power allocation and dimensionality. When not carefully designed, it may degrade overall performance. Simulation results show improvement in SOP by 19% and EE by 76% compared to the second scheme (beamforming with RIS (fixed)), which is the next best model in terms of improved results for the SOP and EE as listed in Table 14.

**Figure 6:**
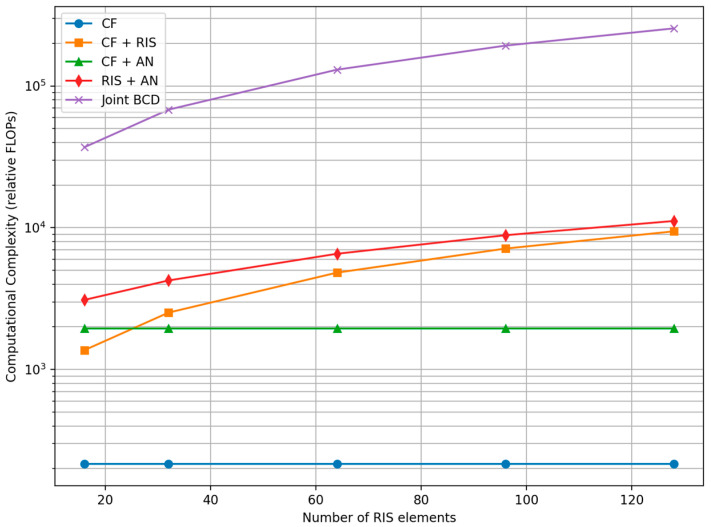
Computational Complexity vs. RIS Size.

**Figure 7:**
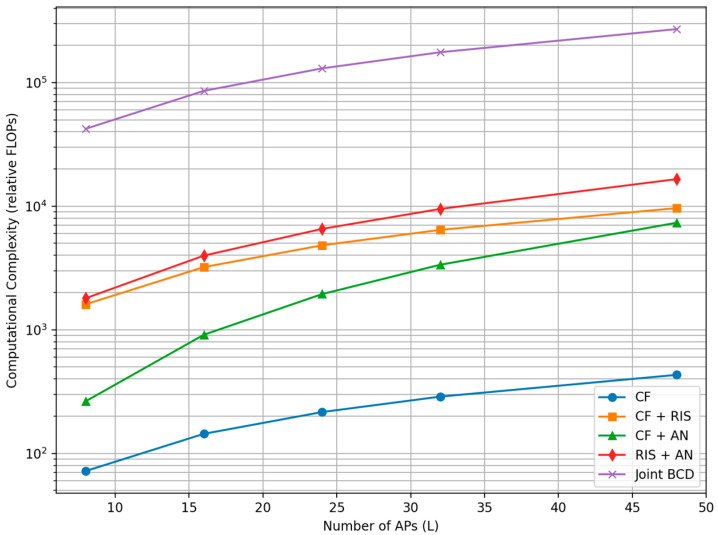
Computational Complexity vs. Number of APs.

Figure 6 and Figure 7 compare the computational complexity of the considered schemes as a function of the number of APs and RIS elements based on the complexity equations defined in Table 13. The baseline CF scheme exhibits the lowest complexity. RIS-assisted schemes incur an additional cost due to cascaded channel construction. The proposed joint BCD-based RIS and AN optimization introduces higher complexity due to iterative greedy phase updates; however, the number of BCD iterations is small, resulting in a manageable complexity increase.

### 5.6. Discussion

Simulation results show that the baseline CF yields very low secrecy rates, confirming the weakness of purely distributed beamforming without PLS enhancements. Adding RIS (CF with RIS) improves rates moderately. Adding AN only (CF with AN) protects against eavesdropping but degrades multi-user performance, giving mixed results. The proposed RIS with AN scheme (RIS with AN) yields a significant right-shift in the CDF, demonstrating reduced eavesdropper advantage via the RIS-induced channel shaping. Joint BCD (Joint BCD) achieves the best secrecy performance, improving both median and 95%-likely secrecy rates by jointly optimizing all components.

The proposed BCD scheme achieves the lowest SOP, indicating high reliability in maintaining secrecy above the target rate. RIS-only and AN-only designs show partial advantages but are consistently inferior to the joint method.

The proposed joint BCD RIS–AN scheme achieves the highest SR and SOP and improved EE by jointly optimizing precoding, artificial noise, and RIS phase shifts. These performance gains come at the expense of increased computational complexity due to iterative BCD optimization and greedy RIS phase updates. Nevertheless, the required number of iterations is small, and the optimization can be performed over large channel coherence intervals. Therefore, the proposed scheme provides a favorable complexity–performance trade-off, where moderate additional complexity yields substantial secrecy and energy efficiency improvements.

While RIS-based schemes consume additional element power, the joint BCD scheme achieves the highest EE thanks to enhanced secrecy rates, improved channel alignment, and effective AN allocation. This highlights the practicality of intelligently optimized RIS deployment for energy-aware secure communications.

From this case study, the following observations are drawn: RIS, AN, and ZF/RZF must be jointly optimized to realize their full PLS potential. Greedy per-element RIS updates combined with power-split ZF design yield a superior secrecy–EE tradeoff. BCD is effective for non-convex joint RIS–precoder design, outperforming simple fixed or random strategies. Energy efficiency improvements are significant even when accounting for RIS hardware overhead. RIS-aided CFMM is a promising paradigm for secure, low-power 6G access networks.

## 6. Conclusions and Future Work

The growth of CFMM networks marks a significant advancement in wireless communications. However, the inherent characteristics of CF networks, namely their dense deployment, high user mobility, and continuous data exchange, pose significant challenges to existing authentication mechanisms in terms of scalability, latency, and computational burden. This paper discusses different aspects related to the security in CFMM networks. Securing these networks requires a comprehensive and multi-layered approach. This involves leveraging PLS, implementing robust authentication mechanisms, deploying intrusion detection systems, and embracing emerging technologies such as blockchain and quantum cryptography to ensure resiliency against various security threats. Beamforming and precoding enhance resistance by focusing energy on legitimate users. Power control and dynamic clustering mitigate the impact of jamming. Advanced signal processing techniques like pilot contamination mitigation and anti-jamming beamforming ensure secure communication. BCD is an efficient iterative strategy suitable for non-convex joint optimization problems by dividing the problem into subproblems easier to be handled. The proposed joint BCD-based RIS and AN greedy optimization exhibits the most promising performance. The simulation results confirm that jointly optimizing RIS phase shifts and AN power allocation can significantly enhance both the energy and secrecy efficiency of UC-CFMM systems, emphasizing the importance of coordinated design between passive and active PLS mechanisms.

### 6.1. Limitations of the Review and Lessons Learned

Although this review aims to provide a comprehensive overview of security challenges and PLS techniques for CFMM, several limitations must be acknowledged to ensure transparency and to guide future research.

1. *Scope of Included Literature*: While major IEEE, Elsevier, and MDPI databases have been surveyed, some emerging works, particularly those in pre-print repositories and specialized workshops, may not be fully captured. Additionally, despite a structured process for inclusion and exclusion, the rapid pace of developments in RIS-aided CFMM and near-field XL-MIMO may result in partial coverage of the most recent advances.
2. *Limited Discussion on Hardware Impairments*: Hardware security—e.g., RIS imperfections, AP synchronization, phase noise, mutual coupling, and hardware spoofing attacks—was not covered in the same depth as software- or algorithm-level PLS methods. This remains a critical dimension for future CFMM deployments.
3. *Focus on Downlink Case Study Only*: The presented case study evaluates downlink secrecy performance. The uplink, joint UL–DL optimization, and full-duplex CFMM security aspects are outside the current scope and thus not addressed.
4. *Assumptions on CSI Availability*: Many works surveyed, including the presented case study, assume perfect or partially known CSI. Realistic issues like RIS-induced pilot contamination, RIS calibration errors, and adversarial pilot spoofing remain underexplored.
5. *Limited Evaluation Scenarios in the Case Study*: The simulation framework focuses on representative but controlled scenarios (e.g., fixed user count, single eavesdropper, fixed RIS size). Broader variability—such as multi-eavesdropper threats, near-field conditions, and large-scale RISs—is not fully examined.

Lessons learned from this study include the following points:

- RIS Deployment Can Dramatically Enhance Security—but Only if Jointly Optimized: Random or fixed RIS phase configurations offer marginal gains. The largest secrecy improvements occur when RIS phases, precoders, and AN strategies are jointly optimized.
- AN Alone Is Insufficient: Artificial noise enhances secrecy only when combined with proper beamforming and RIS control. Otherwise, it can degrade user performance more than it harms the eavesdropper.
- Decentralized Processing Demands New Security Models: CFMM’s inherent distribution of APs introduces new vulnerabilities: insecure backhaul, exposed AP hardware, RIS tampering risks, and localization uncertainty for eavesdroppers.
- Secrecy Performance Is Highly Sensitive to CSI Quality: Both legacy CF-MMSE/ZF algorithms and RIS-assisted designs degrade rapidly under imperfect CSI. Robust estimation and pilot protection remain fundamental design pillars.
- Energy Efficiency Must Be Studied Jointly with Security: Security enhancements (e.g., large RIS, AN injection) can raise energy consumption. However, joint optimization can simultaneously increase secrecy and EE, revealing a promising design direction for 6G.

### 6.2. Open Research Challenges and Future Directions

Based on the survey and the presented case study, several key research directions emerge for enabling secure and scalable CFMM architectures in 6G systems.

1. Near-Field and Polar-Domain Modeling for CF–RIS Security: Recent advances in near-field XL-MIMO, polar-domain channel models, and beam squint mitigation have not yet been integrated into CFMM security analysis. Future work should incorporate spherical-wave RIS–user–AP channels, polar-domain precoding for secrecy, near-field AN shaping, and location-dependent eavesdropping models. These aspects will significantly change RIS design and security guarantees.
2. Hardware Security and Physical Validation: Future CFMM systems must account for realistic hardware threats: RIS malfunction, spoofing, or malicious configuration. AP-side hardware impairments (IQ imbalance, clock drift, noise figure variation). low-cost ADC/DAC vulnerabilities. energy-harvesting attackers capable of passive sensing. Testbed validation is necessary to quantify how these impairments affect secrecy rate and SOP.
3. Secure and Scalable CSI Acquisition: State-of-the-art pilot-based CSI estimation is fragile in distributed systems. Future directions include RIS-enhanced pilot authentication, joint channel-and-position estimation for eavesdropper detection, blind or semi-blind CSI estimation resistant to spoofing, and secure feedback via federated or encrypted backhaul. Robust CSI is essential for reliable RIS and precoder optimization.
4. Joint Optimization of RIS, AP Clustering, and User Scheduling: Most existing works keep AP association and user scheduling unchanged. However, in dense CFMM networks dynamic AP clustering, RIS–AP association maps, secure scheduling policies in the presence of eavesdroppers, and resource allocation under secrecy constraints represent major open design problems.
5. Learning-Aided Secure CFMM Architectures: ML and reinforcement learning (RL) provide opportunities to enhance RIS phase selection, adaptive AN generation, secure beam management, eavesdropper identification, and secure AP–UE association. However, ML introduces new vulnerabilities (e.g., model poisoning), so resilient learning frameworks are needed.
6. Joint Security–EE Design Under Practical Power Models: Future studies should consider RIS switching power, amplifier nonlinearities, backhaul power consumption, and AP sleep-control mechanisms to jointly optimize secrecy rate, latency, and power efficiency in real deployments.
7. Multi-RIS and Three-dimensional Passive/Active RIS Security: Emerging trends include stacked RIS layers and hybrid active–passive RIS. Three-dimensional metasurfaces enabling near-field 6G sensing, RIS-enhanced covert communications. These architectures provide new opportunities for secure CF operations but require fresh models and large-scale optimization methods.
8. Investigating quantum-resistant cryptographic algorithms and integrating chaotic maps to prepare for the future advent of quantum computing and other security threats, which could potentially break existing cryptographic schemes.

## Figures and Tables

**Table 1:** The work related to next-generation challenges and requirements.

Year and Ref.	Contributions	Descriptions
2020 [12]	Outlines the vision for 6G wireless communication addressing key challenges (higher capacity, faster data rates, lower latency, and improved security).	Discusses emerging technologies such as artificial intelligence (AI), terahertz communication, blockchain, quantum communication, and cell-free networks.
2021 [13]	Explores the role of CFMM in addressing capacity and coverage challenges focusing on its applications and benefits.	Surveys the design, applications, potentials, and challenges of CFMM in addressing the increasing demands for high spectral efficiency.
2022 [1]	Examines the security issues associated with technologies (AI/ML, SDN, network slicing, and CFMM).	Provides an in-depth survey of 5G and beyond networks, analyzing different performance requirements.
2022 [14]	Exploring the vision, challenges, and key features for 6G IoT systems, proposing a real-time location monitoring system.	Uses Bluetooth Low Energy (BLE) for underground communication applications (for smart homes, smart cities, and energy savings).
2022 [15]	Gives a detailed survey on security in next-generation networks.	Offers in-depth evaluation of security risks associated with both 5G and emerging 6G networks.
2022 [16]	Provides an overview of intelligent AI/ML-driven systems for both current and future use cases extending beyond 5G (fifth generation) (B5G) networks.	Covers the characteristics and challenges of conventional AI/ML in recent ML applications, deployment and standardization efforts, and future trends.
2022 [17]	Discusses the security, privacy, and trust challenges of 6G networks. AI/ML layers, security issues.	Explores technologies such as AI, quantum cryptography, THz communication, and blockchain.
2022 [18]	Explores 6G enabling technologies like AI, blockchain, network slicing, and CFMM for Intelligent Transportation Systems and Smart Cities.	Emphasizes the importance of ensuring security and privacy in the evolving Internet of Vehicles.
2023 [19]	Explores the paradigm of CFMM systems in next-generation mobile networks, examining deployment architectures, application scenarios, and practical challenges.	Proposes techniques such as predictor antennas, hierarchical cancelation, rate-splitting, and dynamic clustering, outlining key research directions for further advancement.
2023 [20]	Discusses the healthcare system’s challenges, particularly in terms of security and privacy, for remote access to data.	Proposes a healthcare environments model highlighting recommended solutions for improving security, privacy, and trust, considering various limitations.
2024 [8]	Explores the design of AI/ML for Beyond 5G (B5G) and 6G networks covering topics like RF chain reduction, pilot contamination, CF MIMO, and security.	Introduces emerging concepts such as AI-enabled beamforming, reconfigurable surfaces, and Terahertz (THz) spectrum use.
2024 [21]	Discusses the signal transmission challenges of limited transmission distances in THz frequencies.	Uses CFMM contiguous AP assignments improves both spectral and energy efficiency.
2024 [22]	Analyzes various cyberattacks on software-defined smart grids (SD-SGs), including distributed denial of service (DDoS), software-defined networking (SDN) controller, and grid-balancing attacks.	Provides an overview of defense strategies and identifies unresolved challenges such as attack recovery, data privacy, and system dependability.
2024 [23]	Highlights the significance of fuzzy logic algorithms in trust evaluation for secure and reliable 6G networks.	Integrating AI and blockchain technologies alongside fuzzy logic ensuring scalability, resilience, and intelligence for future communication networks.
2024 [24]	Comprehensive technology survey on CFMM networks, insights into design challenges, and potential industrial applications.	Provides a survey on 6G’s specifications, applications, and enabling technologies.
2025 [2]	Identifies future research areas along with potential applications in industrial and healthcare sectors.	Explains privacy concerns and future use cases such as virtual reality and smart healthcare.

**Table 2:** Relevant 3GPP standards for co-located MIMO, cell-free massive MIMO (CFMM), and small cell networks.

Network Type	Core 3GPP Concept	Key Enabling Standards	Primary 3GPP References
Cellular (Conventional MIMO)	Cell-Centric Architecture	LTE (4G) and New Radio (NR) (5G) Air Interface	Technical Specification (TS) 36.xxx (LTE), TS 38.xxx (NR), TS 23.501 (5GC)
Cell-Free	Multi-TRP/Coherent Joint Transmission and Centralized Unit/Distributed Units Split	Joint Transmission and Fronthaul	Technical Report (TR) 38.829, TS 38.401, Multi-TRP features in TS 38.2xx/3xx
Small Cell	Low-Power Node in Heterogeneous Networks (HetNet)	HetNet Management, IAB	TR 36.932, TR 38.874, TS 38.401

**Table 3:** Comparison of CFMM, conventional MIMO, and small cell networks.

Aspects	Conventional MIMO	CFMM	Small Cells
Architecture	Centralized BS with many antennas [28]	Distributed APs coordinated by a central processor [13]	Densely deployed low-power BS [36]
Eavesdropping Risk	Moderate, focused on a centralized channel	High due to distributed APs and lack of centralized control	Each cell can serve as a new attack vector
Pilot Contamination	Present but manageable with coordination	Significant due to user-centric clustering and reuse	Severe with dense deployment and reuse
Inter-cell Interference	High, especially at cell edges	Low due to AP cooperation	Moderate to high, depending on frequency planning
Delay (Latency)	Moderate (depends on load/distance; reduced by edge computing)	Potentially lowest (macro diversity, minimal handover delay)	Very low (due to proximity); improved with Integrated Access and Backhaul (IAB)
Secrecy Rate Performance	Moderate, limited by static deployment	High with proper coordination and precoding	Variable: can be high if interference is controlled
Unique Security Challenges	Limited spatial diversity at edges, beamforming vulnerabilities, and key leakage risks [37]	Scalability of AP coordination, synchronization among APs, and secure fronthaul communication	Handoff security, device im-personation, and edge-based attacks [36,38]
Proposed Security Solutions	Artificial noise injection, channel-based encryption, adaptive beamforming, and antenna selection	PLS techniques, authentication mechanisms, secure handover, intrusion detection, and secure SDN	Identity-based encryption, network slicing isolation, blockchain key distribution, and edge AI intrusion detection [39]
Deployment Complexity	Moderate (centralized control)	High (distributed coordination, ultra-dense fronthaul, tight synchronization, and backhaul demands)	Low to moderate (depends on cell density); requires dense IAB planning and site acquisition
Effect of Improving the EE	Enhancing security may reduce EE due to extra power use; careful resource allocation needed	Optimizing EE may increase susceptibility to attacks; power control mechanisms required [40]	Often inefficient; balancing EE and security needs careful planning; lower power limits eavesdropper range

**Table 4:** Eavesdropping vs. Jamming/DoS vs. Active Pilot Contamination.

Aspect	Eavesdropping Attacks	Jamming and DoS Attacks	Active Pilot Contamination Attacks
Nature of Attack	Interception of communication (passive or active).	Disruption by injecting interference or excessive traffic.	Injection of false pilot signals during training phase
Primary Goal	Steal information without detection (passive) or manipulate communication (active).	Degrade reliability, availability, and service quality.	Corrupt CSI estimation to degrade beamforming and enable eavesdropping
Attack Method	Passive listening; active signal manipulation.	High-power interference during data/pilot transmission; flooding channels.	Transmitting deceptive pilots to mislead AP channel estimation
Impact on CFMM Networks	Exploits AP proximity and decentralized structure; threatens confidentiality.	Disrupts multiple AP–user links; more effective if multiple APs are targeted.	Severe degradation of CSI accuracy; leads to performance loss and information leakage
Affected Phases	Data transmission phase.	Both pilot and data transmission phases.	Training phase and data transmission (due to corrupted CSI).
Level of Threat	High, especially undetectable passive attacks.	High, causes service outages and performance degradation.	Very high, directly compromises secrecy and spectral efficiency
Detection Difficulty	Passive: very hard; Active: moderate.	Moderate to high due to distributed APs.	High; requires statistical tests or machine learning.
Mitigation Approaches	Encryption, beamforming, PLS techniques.	Anti-jamming strategies, spatial diversity, power control.	Robust pilot design, spoofing detection tests, secure channel estimation.
Relevance to CFMM	Decentralized architecture increases vulnerability.	Many APs equal more attack surfaces but also spatial resilience.	Lack of AP coordination enables wide-spread contamination.

**Table 5:** Beamforming and secure precoding in CFMM networks.

Year	Secure Scheme and Contributions	Limitations
2020	Optimal beamforming with power optimization [11]—Derives SC bounds under imperfect CSI, pilot contamination and AP density	High computational cost. Limited user mobility. Overlooks AP synchronization, fronthaul limits, and power constraints.
2020	Beamforming and resource allocation strategies [48]—Derives analytical expressions for harvested energy, ergodic SR, and convex power-control formulation.	Ideals backhaul and simplified channel assumptions. Neglects realistic backhaul constraints, multi-user fairness, and channel correlations.
2021	Decentralized coordinated beamforming using unsupervised learning [90]—Reduces coordination overhead and computational complexity.	Convergence issues in many environments. Generalization gap and hardware constraints. Delays in large-scale setups.
2021	Enhanced Normalized Conjugate Beamforming for CFMM networks [85]—Achieves better SE, lower interference, and improved scalability. Refined power normalization for fairer power distribution.	CSI dependency, quasi-static channels, neglects high-mobility and fast-fading users. Ignores fronthaul/backhaul limits.
2022	Hybrid beamforming with matching theory-based antenna selection [82]—Improves SE/EE under realistic channels.	Matching theory scalability issues. Hardware, mobility, and imperfect CSI constraints.
2023	Secure Precoding for dynamic clustering [52]—Enhances EE, scalability, and robustness. APs adjust beamforming dynamically.	High complexity and hardware constraints. Increased signaling overhead and scalability limits.
2023	Low complexity precoding/decoding techniques [91]—Reduces signaling overhead and improves robustness to CSI errors.	High computational cost for IoT. Limited validation and scalability under mobility.
2023	Enhanced beamforming design in multi-user CFMM [92]—Optimizes SE–security trade-off. Minimizes eavesdropper leakage effects.	CSI imperfections, hardware constraints, simplified eavesdropper models.
2024	Partial ZF (PZF) precoding and power optimization [93]—Path-following algorithm with greedy AP selection.	Single eavesdropper assumption. Simplified real-world channels and scalability challenges.
2024	Beamforming optimization minimizing transmit power with SINR constraints [94]—Avoids centralized overhead.	CSI uncertainty, signaling overhead, and hardware synchronization limits.
2024	Optimal antenna design, beamforming, and power allocation via DNN [95]—Demonstrates practical improvements.	High computation. Ignores real-world impairments and power trade-offs.
2024	GNN-based beamforming and resource allocation [83]—Reduces computation and enhances SE and QoS.	Training complexity, lack of transparency, generalization and latency issues.
2024	Joint Beamforming and Backhaul Optimization with dynamic AP clustering [84]—Improves scalability, EE, and SE.	Hardware limits. Complexity in dense networks. Mobility and CSI robustness issues.
2024	Joint beamforming and power-splitting (PS) for CFMM/small cells [96]—Low-complexity iterative design with EH bounds.	Ignores real channel estimation errors and nonlinear EH effects.
2024	Cooperative hybrid beamforming for CF massive MIMO—wave networks [97]—Improves SE while reducing signaling.	Channel estimation complexity. Hardware and inter—AP interference issues.
2025	Antenna selection and precoding via decentralized ML [98]—Improves SE, reduces fronthaul load.	Training complexity, coordination overhead, limited adaptability.
2025	ML-based AP selection in user-centric CF networks [99]—Improves load balancing, SE, and QoS with lower computational cost.	Dependent on training data. Generalization, latency, and ML energy cost trade-offs.

**Table 6:** Artificial noise (AN) injection schemes for CFMM networks.

Year	Secure Scheme and Contributions	Limitations
2023	AN with beamforming nulling and resource allocation to optimize sensing and communication [80]. Integrates sensing and communication for user-centric CF networks. Proposes dual-function APs for legitimate user transmission and environmental sensing. Ensures security against both information and sensing eavesdroppers. Formulates a trade-off between secrecy rate, sensing accuracy, and power constraints.	High computational complexity and perfect/known eavesdropper CSI assumption. Active sensing eavesdroppers not considered. Degraded sensing due to sensing–communication trade-offs. Practical deployment challenges with fronthaul signaling, hardware impairments, and lack of real-world validation.
2023	AN injection with a tractable analytical model using stochastic geometry [103]. Derives closed-form SR expressions for spatially distributed APs and eavesdroppers. Enables scalable deployment and demonstrates superior secrecy over classical cellular massive MIMO.	Assumes ideal propagation and passive eavesdroppers, ignoring adversarial knowledge. Complex computation, fronthaul limits, and pilot contamination. No experimental validation.
2023	Beamforming and AN technique with power optimization in CFMM downlink phase [104]. Investigates imperfect CSI on PLS. Derives closed-form SRs considering estimation errors and pilot contamination. Demonstrates robust secrecy at design level.	Assumes statistical CSI eavesdroppers and ignores active attacks. Joint beamforming/AN optimization complex for real-time large-scale deployments. No experimental validation. Limited CSI robustness.
2024	AN-aided beamforming strategy combining beamforming and AN to enhance secrecy [105]. Optimally allocates power between signal and AN. Robust under eavesdropper CSI uncertainty with simulation validation.	Assumption of partial/statistical CSI. High computational complexity. Limited real-time applicability and scalability. Focus on single eavesdropper.
2025	AN injection with adaptive power allocation [101]. Derives theoretical bounds and achievable SRs under distributed AN for 5G. Evaluates efficiency, scalability, and computational feasibility.	Relies on passive eavesdropper models and ignores colluding attackers. Not fully realistic under real-world variations. Overlooks fronthaul limits and hardware non-linearities.

**Table 7:** RIS-aided CFMM networks.

Year	Secure Scheme and Contributions	Limitations
2020	Precoding, AN, and intelligent reflecting surfaces (IRS) [111]. Joint optimization of key variables with closed-form updates.	Perfect CSI assumption and non-convexity. Computational complexity. Hardware and practical model limitations.
2021	RIS-Aided Multi-User Communication [107]—Provides a comprehensive overview for RIS-assisted systems, highlighting improvements in SE, EE, and interference management in multi-user scenarios.	Ideal RIS hardware and assumptions. Limited real-world validation. Lacks consideration of mobility or time-varying channels.
2022	RIS-assisted CFMM over spatially correlated channels [108]. Incorporates spatial correlation into optimization. Demonstrates correlation effects on system scalability and performance degradation.	Simplified correlation structure. High CSI acquisition complexity. Ignores RIS impairments (e.g., quantized phase shifts, coupling). Mobility constraints.
2022	Joint Beamforming and RIS Optimization [112]. Enhances secrecy rate and robustness against jamming.	Perfect CSI assumption, computational cost, and hardware scalability constraints—Overlooks scalability and RIS hardware impairments.
2022	RIS-aided CFMM with joint beamforming [113]. Demonstrates enhanced SR compared to non-RIS and CF systems.	Assumes passive eavesdroppers and perfect CSI. High computational.
2023	Hybrid reflecting/transmitting IRS (HR-RIS) CFMM with active relaying and passive reflection [59]—Outperforming hybrid and conventional RIS. Superior SE at low SNR regions for active/passive processing.	Increased complexity from joint active/passive components. Perfect CSI and estimation feedback. Scalability and hardware limitations.
2023	Machine Learning-based approach for RIS-enhanced CFMM networks [109]—Reduces computational cost with ML dynamic adaptation. Demonstrates SE and EE improvements.	Generalization limitations in unseen channels. Coordination overhead for real-time learning between APs. Scalability constraints.
2023	Cooperative active RIS for secure communication [114]—Joint optimization reduces feedback overhead and improves SR in dense deployments.	Hardware constraints. Mobility limitations. Scalability challenges in large systems.
2024	Multi-RIS and AN enhanced CFMM. Dual-tier mitigation design addressing both jamming and eavesdropping [37]—Demonstrates SR and anti-jamming improvements. Manages multiple RISs in large-scale CF networks.	Complexity concerns. Unrealistic assumptions of full attacker knowledge. Hardware limitations. Mobility constraints.
2024	RIS-aided CFMM for 6G networks [106]—Explores ultra-reliable communications and smart city use cases. Evaluates trade-offs between cost, complexity, and performance, highlighting design insights.	Channel estimation complexity. Hardware constraints (phase-shift precision, scalability). Mobility and dynamic variations. Real-world validation absent.
2024	Simultaneously transmitting and reflecting RIS (STAR-RIS) scheme [115]—Formulates and maximizes SR under dual reflection/transmission. Jointly optimizes AP active beamforming and RIS coefficients.	Complexity and mobility limitations. Relies on perfect CSI across all links. Hardware constraints.
2024	RIS-aided CF network design against multiple eavesdroppers with imperfect CSI [9]—Novel optimization to maximize SE under imperfect CSI. Joint design for RIS phase shifts, transmit power, and AN covariance to enhance robustness.	Computational complexity. Bounded error model may not capture all impairments. Practical RIS constraints and scalability concerns.
2025	Multi-active RIS-assisted CFMM in THz communication [116]—Joint optimization for precoding and reflection coefficients, achieving SE–EE trade-offs under THz constraints. Comprehensive evaluation provided.	High computational and scalability limits. Perfect CSI assumption. No modeling of nonlinearities or amplifier noise. Mobility and beam split effects at THz frequencies not addressed.
2025	Optimal reconfigurable intelligent surface deployment [117]—Uses different search methods for RIS placement and phase shifts.	Single eavesdropper and idealized deployment. Scalability issues, static assumptions. No multi-RIS or dynamic network adaptation.Finds near-optimal RIS locations under constraints.

**Table 8:** Authentication and key management approaches in CFMM systems.

Year	Secure Scheme and Contributions	Limitations
2020	Physical layer authentication (multicast beamforming) [134]. Distributed signal processing eliminates the need for full CSI exchange. Introduces ML-aided power control, user-centric dynamic AP clustering, and soft handover. Enhances interference characterization and multicast beamforming efficiency.	Assumes perfect clock synchronization and TDD reciprocity among APs. High computational cost. Fronthaul capacity constraints. Limited consideration of mobility and hardware impairments.
2021	Channel-based key extraction using pilot randomization [133]. Proposes countermeasures against active attacks and quantifies their impact on key generation rate and reliability. Validates defense effectiveness through synthetic and real-world scenarios.	Increased computational complexity. Sensitive to channel dynamics. Hardware-specific vulnerabilities and implementation issues not fully addressed.
2023	Multifactor authentication protocol based on Elliptic Curve Diffie–Hellman (ECDH) [45]. Lightweight mutual authentication integrated with blockchain using proof-of-stake consensus. Robust against eavesdropping, replay, and man-in-the-middle attacks in high-mobility environments.	Proof-of-stake latency and overhead. Simplified threat model. Lack of large-scale field validation with real blockchain throughput and deployment evaluation.
2024	Joint optimization framework for assigning Monitoring Nodes (MN) [125]. Derives analytical expressions for Monitoring Success Probability (MSP) under Maximum Ratio (MR) and Partial Zero-Forcing (PZF) schemes. Enables efficient tractable algorithms and improved load balancing.	Mode assignments rely on statistical CSI, ignoring fast channel variations. High computational cost and signaling overhead. No experimental validation. Limited to static or distributed wired links.
2024	Blockchain-based technology for authentication [153]. Enables dynamic spectrum trading within CFMM via blockchain. Incorporates Stackelberg game-driven pricing and smart contract implementation for secure transactions.	Idealized network and traffic assumptions. Lack of blockchain latency and throughput validation. Simplified operator behavior. No real-world or large-scale evaluation.
2024	CSI acquisition in CFMM surveillance systems [159]. Addresses large-scale distributed CSI collection challenges. Reduces pilot contamination and improves detection accuracy. Demonstrates improved tracking through enhanced channel estimation.	High computational complexity. Sensitivity to mobility. Limited real-world validation and pilot resource constraints.

**Table 9:** Intrusion Detection Systems (IDS) in CFMM networks.

Year	Secure Scheme	Contributions	Limitations
2019	Spoofing detection via minimum-description-length (MDL) [53]	Secure multigroup multicasting in CFMM systems under pilot spoofing attacks using distributed conjugate beamforming with normalized power constraints at APs. Closed-form secrecy performance metrics and ergodic secrecy rate evaluation.	Use of MDL detection only. Restricted channel/beamforming models. Multicast-only and static scenarios. Lack of real-world validation.
2020	Detection and mitigation of multiple active eavesdroppers through joint AP selection and power control [126]	Supports multiple eavesdroppers with joint AP selection and power control. Low-complexity algorithm achieving strong performance gains.	Sub-optimal algorithm. Assumes ideal channel models. Computational overhead at large scales.
2021	Channel-based spoofing attack detection scheme using channel virtual representation (C-VR) in UAV/WAVE–MIMO 5G networks [59]	Introduces Principal Components of Channel Virtual Representation (PC-CVR) as a new channel fingerprint capturing spatial beam characteristics. Dual detection frameworks provide significant detection improvements.	Limited attacker sophistication (less effective against adaptive spoofers). Depends on stable channel statistics. Simplified evaluation setup. Single-layer NN architecture.
2023	Pilot spoofing detection [161]	Analytical outage expressions under pilot contamination considering imperfect CSI. Derives exact multi-fold integral expressions for OP without pilot contamination. Validates analytical results against simulations for OP and ergodic rate metrics.	Accuracy depends on moment-matching quality. Assumes Rayleigh fading and MRC combining (excludes complex channel models). Neglects fronthaul/backhaul limitations, mobility, and hardware impairments. Static pilot assignment.
2024	Jamming detection for multi-user beam training under random pilot jamming in uplink phase [164]	Effective detection under general spatial correlation scenarios. Includes analytical performance characterization and two-step MMSE channel estimation. Strong simulation results under spatial correlation.	Single-jammer assumption. Pilot-based attack model only. UAV/WAVE–MIMO specificity limits applicability. Relies on known spatial correlation models. No protocol-level integration.
2025	Multi-layer intrusion detection with PLA [165]	Integrates PLA with UAV/WAVE systems. Optimized model generation and discussion of emerging challenges in 6G cybersecurity.	Not yet applied explicitly on CFMM networks. Lacks real-world validation. Does not address adversarial effects on learning mechanisms.

**Table 10:** Secure handover mechanisms in CFMM networks.

Year	Secure Scheme	Contributions	Limitations
2021	Beamforming and handover mechanisms [167]	Integrated mobility-aware UAV/WAVE channel model and dynamic user-centric handover. Beamforming and dynamic user association techniques reduce unnecessary handovers and mitigate mobility and channel aging effects.	Idealized channel knowledge assumptions. Lack of real-world validation. Limited to downlink and single-user scenarios. High computational complexity and latency cost in dense AP deployments.
2022	CPU cluster and AP handover rate in scalable CFMM with hybrid AP selection [166]	Closed-form handover rate derivations and inclusion of handover delays in a mobility-aware SE model. Quantifies performance impacts and compares with distributed MIMO.	No real-world trials. Single-user mobility and fixed AP clustering. Ignores uplink channel aging and estimation errors.
2023	Distributed soft handover with imperfect CSI [169]	Reduced AP and pilot reconfigurations with low computational complexity and massive access support.	Simulation-based validation only. Downlink-only with ideal coordination. Neglects overhead, mobility, and latency.
2023	Distributed learning-based power control and mobility management schemes [171]	Introduces the radio stripe concept and identifies deployment challenges. Proposes integration with smart infrastructure for real-world feasibility.	Lacks quantitative evaluations. Conceptual-level operation only. Undefined scalability and untested issues like aging, interference, and environmental variation.
2023	Handover in UC-CF network with varying AP cluster strategies [176]	Analyzes cluster handovers in single-user CFMM networks. Evaluates handover probability, rate, and user throughput. Proposes strategies to reduce signaling overhead and handover frequency.	Single-user scenario; ignores multi-user and interference effects. Ideal channel assumptions. Limited mobility consideration. Fixed cluster formation without dynamic reconfiguration.
2023	Handover process in CF networks [177]	Provides real-world experimental analysis showing that CF architectures reduce handover frequency and improve connection stability. Offers guidelines for optimizing handover strategies in future systems.	Does not capture large-scale network dynamics. Hardware constraints present. Focus on downlink only. Limited consideration of highly mobile users.
2024	CPU-cluster and AP handover [168]	Derives closed-form handover rate expressions and mobility-aware throughput models. Compares AP-selection schemes and demonstrates high-percentile user benefits.	No hardware trials or field tests. Single-user mobility assumption. Neglects channel aging and estimation errors. Simplified control-plane modeling and fixed clustering.
2024	HandOffs (HOs) in user centric CFMM networks [170]	Models handoffs (adding/dropping APs) in user-centric CFMM using a partially observable Markov decision process (POMDP). Captures temporal channel evolution and uncertainty. Proposes mobility-aware handover reduction mechanism.	Simulation-only validation and single-user focus. Static AP density assumption. High complexity vs. practical deployment. Relies on accurate channel models, fixed clustering strategy.
2025	Low-complexity active eavesdropper detection with large-scale fading estimation [172]	Models multi-eavesdropper scenarios with joint AP selection and power optimization. Performs eavesdropper detection with large-scale fading estimation, showing significant performance gains.	Single-eavesdropper-per-user assumption. Complex mixed-integer optimization. Perfect CSI assumption for legitimate links. Downlink-focused simulation without hardware validation.

**Table 11:** A summary of key papers on secure SDN-CFMM.

Year	Research Focus Area	SDN/Virtualization Relevance
2021	Trust-based access control via SDN [183].	O-RAN/SDN control-plane design suitable for securing distributed CF architectures under O-RAN (implicit).
2021	CFMM survey including network architectures, fronthaul/control signaling, and future technologies like virtualization and SDN [28].	Discusses virtualization and control-plane separation.
2021	Decentralized beamforming and power control for CFMM [90].	SDN-enabled coordination and centralized/decentralized control frameworks.
2022	Deep learning application reviews in CFMM for channel estimation and resource allocation [184].	Reinforces ML–SDN synergy in CF systems.
2022	Power control with artificial noise across distributed APs [98].	Coordination by the network controller (implicit).
2022	Multi-antenna AP secrecy analysis [40].	Central power control (implicit).
2023	Energy-aware orchestration in CFMM [86].	Demonstrates SDN/NFV-managed resource control, capturing the essence of end-to-end orchestration in CF systems.
2023	PHY-layer support for CFMM in O-RAN [185].	Maps CFMM into O-RAN’s SDN/NFV control architecture and network slicing frameworks.
2023	Mobility challenges in CFMM [19].	Highlights scenarios where centralized SDN control could improve adaptability.
2023	SDN-controlled RIS-assisted CFMM including coordinated reflections and dynamic path selection [186].	Integrates SDN orchestration for PLS. The SDN controller manages multi-beam routing to avoid eavesdroppers and adapt to user mobility.
2024	Deep learning-based channel estimation in CFMM [187].	Enables data-driven orchestration control via SDN.
2024	Ultra-dense CFMM overview [33].	Highlights orchestration challenges for SDN adoption.
2024	Central control of RIS-assisted CFMM combining multiple RIS elements and artificial noise with AP selection [37].	Centralized control for phase shift and AN coordination; supports frameworks where SDN coordinates nodes.
2024	Active eavesdropper detection and mitigation [93]	Relates to AP selection and control clustering.
2024	Control-plane design for future wireless systems [188].	Deep learning powered SDN/NFV security architecture (implicit).
2024	Power control across CFMM nodes [189].	Uses graph neural networks for distributed power control with architectures suitable for SDN controllers.
2024	Future CF–UAV/WAVE frameworks including potential SDN-based scenarios [190].	Describes flexible architectures enabled by SDN.
2025	SWIPT in CFMM using stacked intelligent UAV/WAVE [191].	Long-term CSI and cloud-driven optimization suggesting SDN orchestration opportunities.
2025	Cross-layer SDN/NFV/AI security framework [182].	Supports a cross-layer security framework for network slicing.

**Table 12:** Simulation parameters and configuration settings.

Notation	Definition
K, L, N	Number of UEs, APs, and antennas per APs.
eve	The eavesdropper.
N_RIS_	The total number of RIS elements.
k, i	UEs Index.
l, j	APs Index.
(·)*, (·)^T^, and (·)^H^	Conjugate, transpose, and Hermitian transpose.
$H_{kl}$∈ C^N^	Direct AP-UE channel.
$H_{ap-ris}$	AP-RIS channel.
$H_{ris-u}$	RIS-UE channel.
$H_{ap-eve}$	Direct AP-Eve channel.
$H_{ris-eve}$	RIS-Eve channel.
P_total	The total power.
P_data	The data transmitted power.
P_AN	The AN power.
| · |	Scalar absolute value.
**‖** · **‖**	Vector norm
${\left[x\right]}^{+}$	$\max\left(0,x\right)$
{·} and Var{·}	Expectation and variance of a random variable.
δ	eve’s beamforming scaling factor = 0.25
$\sigma^{2}$	The noise variance = 1 × 10^−3^
z∼CN (0, C)	Random vector of complex Gaussian random variables (zero mean and covariance matrix C).
$Q_{an}$	The AN covariance matrix where the AN vector $V_{an}∼CN(0,Q_{an})$
α	The pathloss exponent (3.5 for AP-UE channel (NLOS), 2.8 for AP-RIS and RIS-UE channels).
_I_	number of BCD iterations
$P_{RIS}$	number of RIS phase candidates
$P_{greedy}$	number of greedy passes

**Table 13:** Complexity per operation and per scheme.

Operation	Complexity	Scheme	Complexity
ZF precoder (pseudo-inverse)	$O(LK^{2})$	CF (no RIS, no AN)	$O(LK^{2})$
RIS cascaded channel	$O(LN_{RIS}K)$	CF + RIS (fixed)	$O(LN_{RIS}K+LK^{2})$
Artificial noise (null space)	$O(L^{2}K)$	Artificial noise (CF + AN)	$O(LK^{2}+L^{2}K)$
SINR+ secrecy evaluation	O(LK)	RIS + AN + PH (modeled)	$O(LN_{RIS}K+LK^{2}+L^{2}K)$
Greedy RIS update	$O(LK^{2})$	Joint BCD RIS + AN	$O(I(LN_{RIS}K+LK^{2}+L^{2}K+P_{greedy}N_{RIS}P_{RIS}LK))$

**Table 14:** SOP and EE of various secure schemes.

Scheme	SOP	EE (Bits/Joule)
Beamforming only	0.9300	0.001950
Beamforming with RIS (fixed)	0.9133	0.002465
AN with beamforming only	0.9617	0.001369
Proposed scheme (modeled)	0.9317	0.002368
Joint BCD (improved proposal)	0.7400	0.004396

## Data Availability

The original contributions presented in this study are included in the article. Further inquiries can be directed to the corresponding author.
